# *In vitro* Models of Bone Remodelling and Associated Disorders

**DOI:** 10.3389/fbioe.2018.00134

**Published:** 2018-10-11

**Authors:** Robert Owen, Gwendolen C. Reilly

**Affiliations:** Department of Materials Science and Engineering, University of Sheffield, Insigneo Institute for in silico Medicine, Sheffield, United Kingdom

**Keywords:** osteoblast, osteoclast, co-culture, tissue engineering, 3D cell culture, bone remodelling, *in vitro* model, osteoporosis

## Abstract

Disruption of bone remodelling by diseases such as osteoporosis results in an imbalance between bone formation by osteoblasts and resorption by osteoclasts. Research into these metabolic bone disorders is primarily performed *in vivo*; however, in the last decade there has been increased interest in generating *in vitro* models that can reduce or replace our reliance on animal testing. With recent advances in biomaterials and tissue engineering the feasibility of laboratory-based alternatives is growing; however, to date there are no established *in vitro* models of bone remodelling. *In vivo*, remodelling is performed by organised packets of osteoblasts and osteoclasts called bone multicellular units (BMUs). The key determinant of whether osteoclasts form and remodelling occurs is the ratio between RANKL, a cytokine which stimulates osteoclastogenesis, and OPG, its inhibitor. This review initially details the different circumstances, conditions, and factors which have been found to modulate the RANKL:OPG ratio, and fundamental factors to be considered if a robust *in vitro* model is to be developed. Following this, an examination of what has been achieved thus far in replicating remodelling *in vitro* using three-dimensional co-cultures is performed, before overviewing how such systems are already being utilised in the study of associated diseases, such as metastatic cancer and dental disorders. Finally, a discussion of the most important considerations to be incorporated going forward is presented. This details the need for the use of cells capable of endogenously producing the required cytokines, application of mechanical stimulation, and the presence of appropriate hormones in order to produce a robust model of bone remodelling.

## Introduction

Bone remodelling occurs throughout life and is an essential physiological process that renews the skeleton. It maintains or improves bone strength by replacing primary, immature bone and old, micro-damaged or fractured bone, as well as maintaining calcium homeostasis (Boyce et al., 2012). The resorption and formation processes are balanced, and remodel approximately 5% of cortical and 20% of trabecular bone each year. Whilst the latter accounts for only 25% of the total bone volume, the increased surface area to volume ratio results in a 10 times higher metabolic rate (Fernández-Tresguerres-Hernández-Gil et al., 2006). It is a continuous event throughout life, but the balance between resorption and formation changes. In healthy individuals, formation dominates for the first three decades until peak bone mass is achieved (Kini and Nandeesh, 2012). This bone mass is then maintained for approximately 20 years until resorption begins to outweigh formation and mass declines.

In contrast to remodelling, a homeostatic renewal process where resorption and formation are coupled spatially and temporally, bone modelling defines the shaping or reshaping of bones where osteoblasts and osteoclasts can act independently. This begins before birth and is most apparent during skeletal development and in young adults; however, it still occurs later in life in response to mechanical load (Langdahl et al., 2016; Paiva and Granjeiro, 2017).

### The cellular and molecular basis of bone remodelling

**Figure 1:**
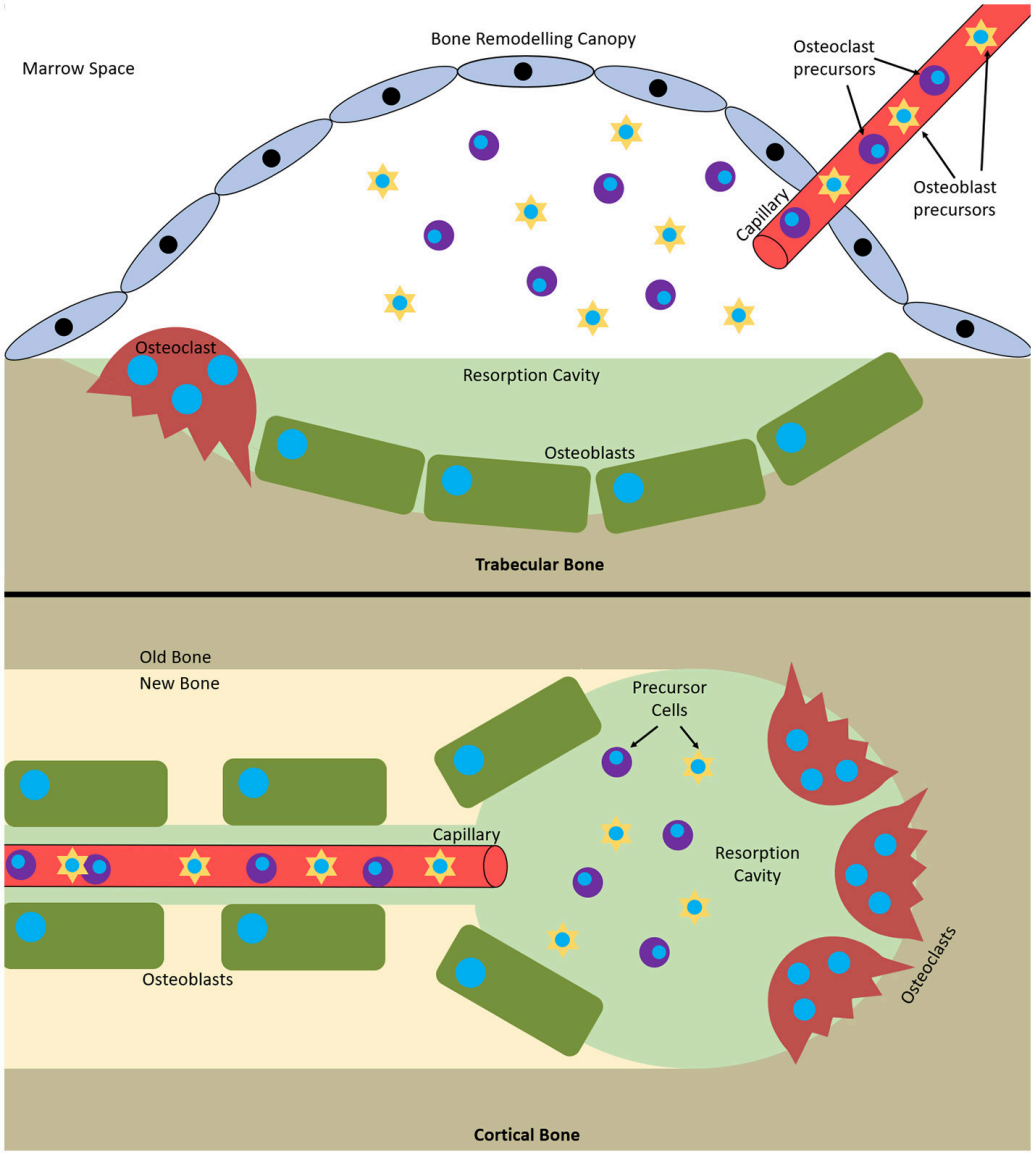
Bone multicellular units in (top) trabecular and (bottom) cortical bone. In trabecular bone they initiate underneath bone remodelling canopies formed from bone lining cells and in cortical bone at points within Haversian canals.

Remodelling occurs via basic multicellular units (BMUs). These are composed from discrete packets of osteoclasts and osteoblasts accompanied by a blood supply and supporting connective tissue (Jilka, 2003). BMUs form and refill tunnels through cortical bone and in trabecular bone they create trenches on the surface. The osteoclasts are at the front, forming the cutting cone or hemi-cone in the case of trabecular BMUs, with osteoblasts behind forming the closing cone or hemi-cone. The BMU can move in all three axes in cortical BMUs and two axes in trabecular BMUs as they are on the surface (Parfitt, 2002; Clarke, 2008) (Figure 1).

Whilst osteoblasts and osteoclasts are directly involved in the bone remodelling process, they are not the only bone cells involved. Osteoblast precursors present in the bone marrow, often referred to as mesenchymal progenitors, mesenchymal stromal cells or mesenchymal stem cells (MSCs), are required to differentiate into osteoblasts and support osteoclast formation (Rucci, 2008; Lindner et al., 2010). Osteocytes are terminally differentiated osteoblasts that become embedded in the bone during formation in pits called lacunae. They are star shaped (stellate) with multiple cytoplasmic extensions (Tanaka-Kamioka et al., 1998) that occupy the canaliculi that connect the lacunae and make contact with other osteocytes, osteoblasts, cells lining the bone surface, and vasculature via gap junctions (Doty, 1981; Dallas et al., 2013). This network of osteocytes forms a complex communication system that enables them to sense and respond to stresses placed upon the bone by releasing paracrine factors that regulate bone remodelling (Santos et al., 2009; Paiva and Granjeiro, 2017).

**Figure 2:**
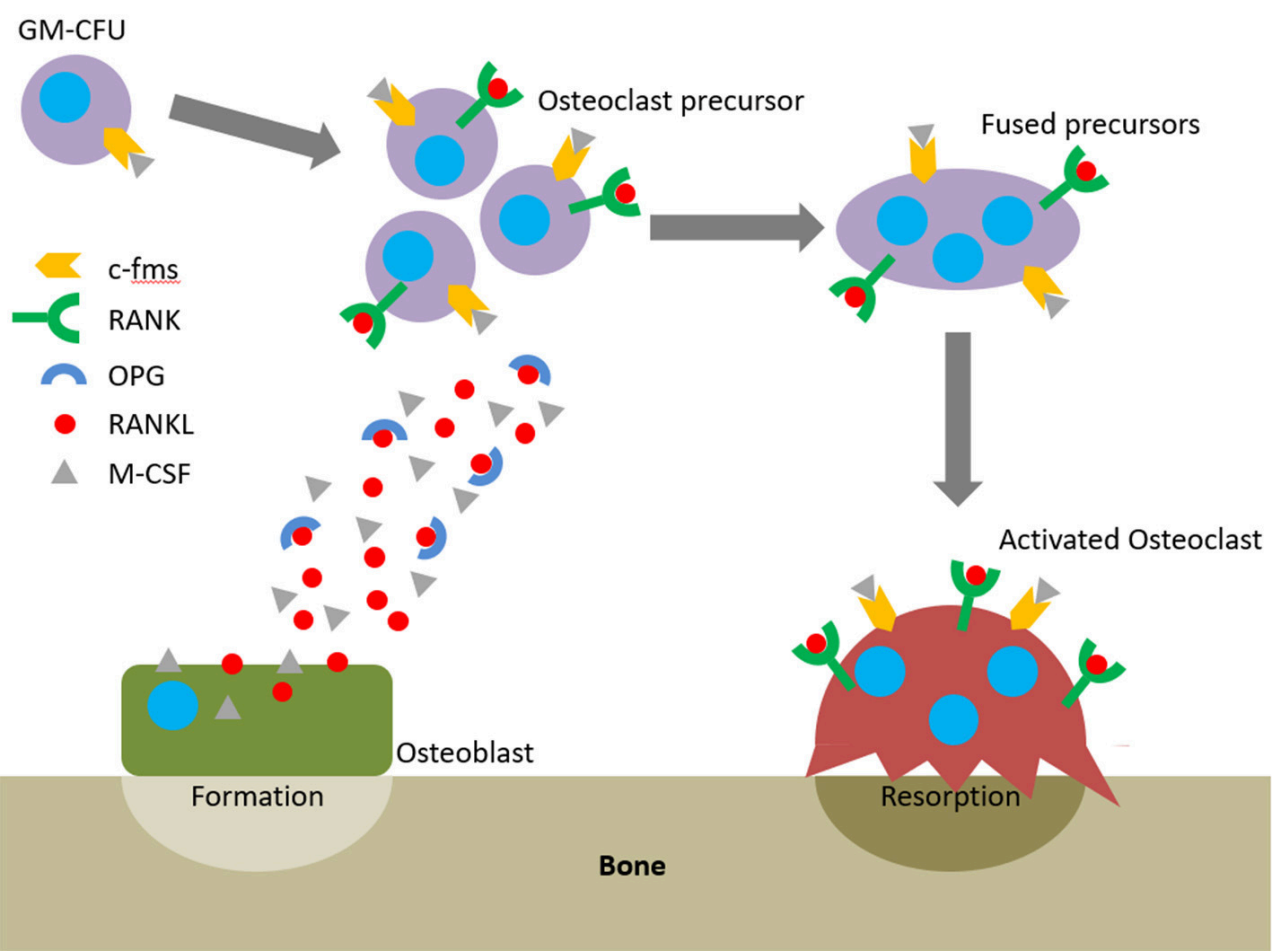
The RANKL/RANK/OPG axis and M-CSF direct osteoclastogenesis and activation.

The action of osteoblasts and osteoclasts within the BMU is tightly coupled via biochemical pathways. Once osteoclast precursors have arrived at the remodelling site from the bloodstream or surrounding marrow, two factors are predominantly responsible for their maturation into osteoclasts: macrophage-colony stimulating factor (M-CSF) and receptor activator of nuclear factor kappa-B ligand (RANKL). These factors bind to their respective receptors on the precursors, colony-stimulating factor-1 receptor (c-fms) and receptor activator of nuclear factor kappa-B (RANK), and initiate osteoclastogenesis. The binding of RANKL to RANK can be antagonised by osteoprotegerin (OPG), a decoy receptor for RANKL that inhibits osteoclastogenesis. Therefore, whether and how much resorption occurs is determined by the RANKL:OPG ratio (Mizuno et al., 1998). Like RANKL, OPG is also produced by osteoblasts meaning that they have a key role in controlling the balance between bone formation and resorption (Figure 2).

Although the RANK/RANKL/OPG axis is perhaps the best characterised mediator of bone remodelling, it is not the only method by which the constituent cells communicate and the process is controlled. For example, bone morphogenetic proteins (BMPs) can induce osteogenic differentiation and modulate osteoclast fate (Valcourt and Moustakas, 2005), Eph/epherin signalling by osteoclasts can promote or inhibit osteoblastic differentiation depending on the ligand type (Matsuo and Otaki, 2012) and growth hormone and insulin-like growth factors can moderate remodelling (Giustina et al., 2008). Recently the role of extracellular vesicles (EVs) in intercellular communication is of particular interest. These can carry multiple types of cargo (e.g., DNA, RNA, proteins, lipids), with bone-related EVs transporting molecules such as BMPs, non-collagenous extracellular matrix proteins such as osteocalcin, RANKL and OPG, and microRNAs that can promote or inhibit osteoblastic differentiation (Xiao et al., 2007; Deng et al., 2015; Liu et al., 2018). This type of signalling has been identified between multiple cell types involved in bone remodelling, including: MSCs and osteoblasts, osteoblasts and osteoclasts, osteocytes and osteoblasts, and osteoclasts and osteoclasts. However, as the precise roles of EV signalling are still being investigated, it is not something that is commonly examined *in vitro* when studying bone remodelling. Therefore, this review predominantly focusses on the roles of RANKL and OPG. Work performed to date on EVs involved in remodelling has recently been comprehensively reviewed by Liu, et al., and Yuan, et al., (Liu et al., 2018; Yuan et al., 2018).

The earliest haematopoietic precursor that can give rise to an osteoclast is the granulocyte-macrophage colony forming cell (GM-CFU). M-CSF is produced by osteoblasts and stromal cells and its activation of c-fms promotes the survival and proliferation of the GM-CFU (Ross, 2006; Hodge et al., 2007). RANKL is expressed by osteoblasts, T cells, and endothelial cells and its conjugation with RANK commits the GM-CFU to the osteoclast lineage. This upregulates key markers such as tartrate-resistant acid phosphatase 5b (TRAP), the osteoclast specific isoform of tartrate-resistant acid phosphatase (Halleen et al., 2000). Continued exposure to both factors stimulates the preosteoclasts to fuse and form a multinucleated cell. Once activated, they bind to the bone surface and secrete hydrogen ions and proteolytic enzymes to degrade the mineral and organic components of bone, respectively, forming a resorption pit (Bull et al., 2002; Kular et al., 2012). Therefore, active osteoclasts can be identified by three features: the presence of TRAP, multiple nuclei, and the capability to resorb bone.

It has recently been discovered that trabecular BMUs are separated from the surrounding bone marrow by a canopy to create a bone remodelling compartment (BRC). These canopies are likely formed by an extension of the bone-lining cells due to their expression of typical osteoblastic markers. The BRC generates a unique microenvironment conducive to paracrine signalling and facilitates BMU formation and function. It allows control over osteoblast-osteoclast coupling and ensures tightly regulated remodelling. BRCs cover practically all resorptive surfaces and over 50% of formative surfaces, indicating that they form as resorption initiates and are closed as formation completes. Capillaries penetrate the BRC and are thought to serve as conduits for the precursor cells needed to form and maintain BMUs as their lifespan is 6 to 9 months, much longer than the constituent cells (osteoclasts 2 weeks, osteoblasts 3 months). Furthermore, pericytes adhering to the walls of this vasculature are also able to undergo osteogenic differentiation (Henriksen et al., 2014). Disruption of the BRC negatively affects bone turnover and can result in uncoupled remodelling, where bone is resorbed without being replaced (Hauge et al., 2001; Parfitt, 2001; Andersen et al., 2009; Eriksen, 2010; Raggatt and Partridge, 2010; Delaisse, 2014; Wesseling-Perry, 2014).

### The stages of bone remodelling

**Figure 3:**
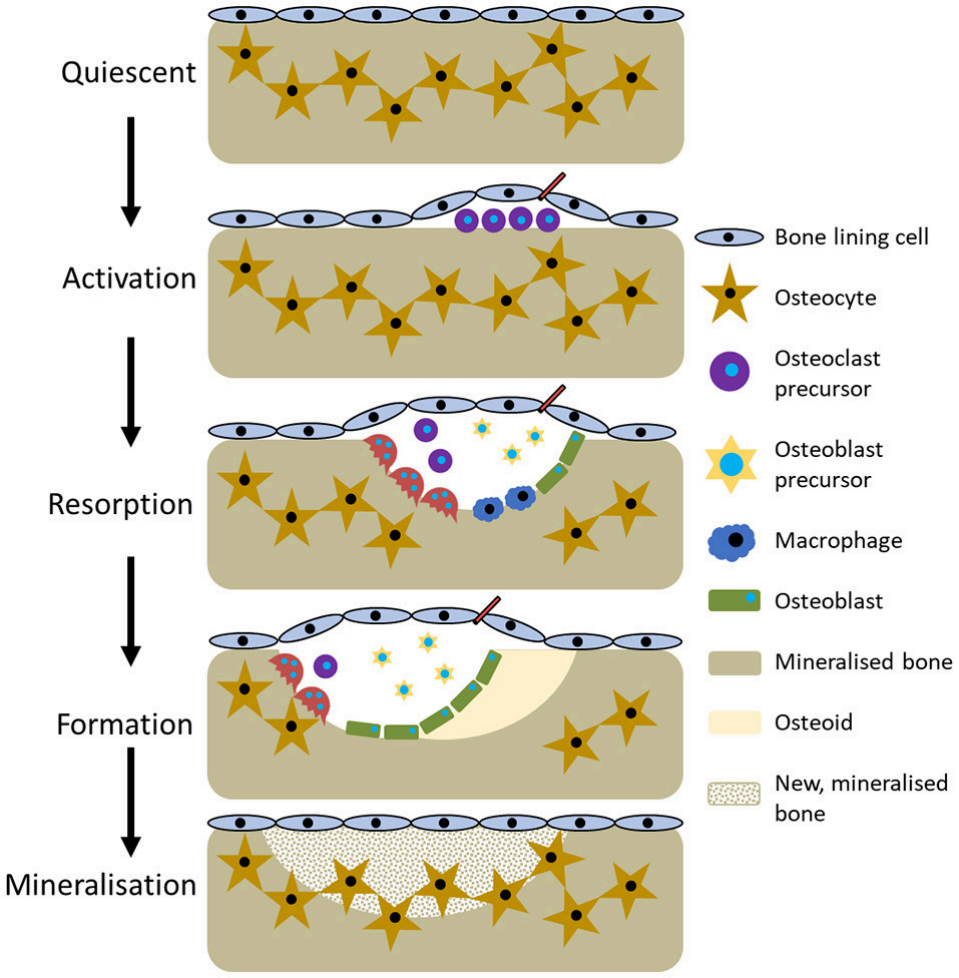
The five stages of bone remodelling.

The current perception of bone remodelling is that it can be divided into five sections, although the nomenclature for each division is not universal. Herein, they will be referred to quiescent, activation, resorption, formation, and mineralisation (Figure 3). Quiescent describes inactive bone prior to remodelling initiation. Then, as a result of events such as microfracture, mechanical loading, or low calcium due to pregnancy or a deficient diet, the activation phase begins. This prepares the bone for remodelling by forming the BRC and recruiting osteoclast precursors, which are subsequently activated by RANKL and M-CSF and attach to the bone surface. Resorption then begins, with osteoclasts degrading the bone and liberating growth factors trapped within the matrix before undergoing apoptosis. Macrophages clear away the debris from the resorption pit and a transition to the formation phases begins. Initially, osteoid, a collagenous matrix, is deposited to fill the cavity. This is mineralised over the following 3–6 months by osteoblasts secreting matrix vesicles that establish an environment conducive to mineralisation by increasing the concentration of calcium and phosphorous ions. During this process, some osteoblasts become trapped and undergo osteocytogenesis whilst others undergo apoptosis or become bone lining cells (Dempster, 2006; Kini and Nandeesh, 2012).

For remodelling to be successful, it is imperative that the existing bone matrix is completely removed prior to the formation of new bone. However, very few collagenases can degrade the fibrillar collagen type 1 found within bone; the most notable exceptions are cathepsin K and matrix metalloproteinase-9 (MMP-9). This difficulty arises from the tightly packed, helical structure of the collagen and surrounding minerals making it inaccessible (Sprangers and Everts, 2017). To overcome this, osteoclasts bind to the bone surface and form a sealed zone through cytoskeleton rearrangement to form a ring of actin. Within this ring the plasma membrane enlarges and becomes convoluted forming a ruffled membrane with finger-like projections to increase surface area and therefore contact with the bone matrix. This compartment is acidified to demineralise the bone matrix, exposing fibrillar collagen within.

This low pH zone also provides the optimal conditions for cathepsin K, a lysosomal cysteine protease secreted through the ruffled border, to degrade the collagenous matrix (Teitelbaum, 2000). The serum concentration of the degradation products of cathepsin K, the carboxy- and amino-terminal telopeptides of collagen type 1 (CTX and NTX, respectively), can be used as markers of bone turnover (Garnero et al., 2003). Similarly *in vitro*, their concentration in culture media can be determined by ELISA as evidence of collagen degradation. MMP-9 is the most expressed MMP by osteoclasts and is also capable of degrading demineralised collagen. However, in addition to this role it also appears to be involved in osteoclast recruitment through the conversion of the inactive form of TNF-α into the active cytokine, promoting osteoclast formation and survival. An extensive review by Paiva and Granjeiro explores the roles of the MMP family in bone (Paiva and Granjeiro, 2017).

### Metabolic bone diseases

Metabolic bone diseases, such as osteoporosis, osteopetrosis and rickets, affect the bone remodelling process. They can alter bone balance both positively and negatively, resulting in either excessive bone formation or bone resorption, respectively. Osteopetrosis is a metabolic bone disease that causes a positive bone balance. It is a rare (1 in 20,000 to 250,000 depending on type) inherited disorder that results in increased bone mass and BMD caused by a dysfunction in the ability of an osteoclast to acidify its resorption pit, resulting in improper resorption (Sobacchi et al., 2013). This impaired resorption combined with continued bone formation results in denser, more brittle bones that are more susceptible to fracture (Tolar et al., 2004; Stark and Savarirayan, 2009).

Rickets is caused by a vitamin D deficiency that inhibits osteoblast progenitors and increases RANKL whilst decreasing OPG expression, resulting in increased bone resorption and turnover (Lips et al., 2013). Multiple myeloma, a cancer of plasma cells, can also cause severe bone destruction through a remodelling imbalance. The hallmark osteolytic lesions form in close proximity to myeloma cells due to hyperactivity of osteoclasts and hypoactivity of osteoblasts (Giuliani et al., 2014).

The most prevalent metabolic bone disorders are osteopenia and osteoporosis, both of which describe a loss of bone mineral density (BMD). The World Health Organisation states that when a patient's BMD falls between 1 and 2.5 standard deviations below that of a healthy young adult they are classed as osteopenic, lower than −2.5 is diagnosed as osteoporotic (National Institutes of Health, 2000). Osteoporosis refers to a group of conditions, rather than a specific, single entity. It is classified as primary type 1, primary type 2 or secondary. Type 1 is the most common and is often referred to as postmenopausal osteoporosis as it is caused by an associated oestrogen deficiency that results in increased RANKL and decreased OPG production and reduced osteoclast apoptosis (Saika et al., 2001; Eghbali-fatourechi et al., 2003; Nakamura et al., 2007). Type II is also known as senile or age-related osteoporosis and can occur in both men and women with age. Finally, secondary osteoporosis refers to when the disorder is present as a consequence of an adverse response to a medication, change in physical activity, or another medical condition. Common examples of this type include glucocorticoid- and immobilisation-induced osteoporosis (Feng and McDonald, 2011), as well as inflammation-induced bone loss as the result of overexpression of RANKL by immune cells during periodontitis or rheumatoid arthritis (Schett and David, 2010; Redlich and Smolen, 2012).

Osteoporosis reduces bone strength through a reduction in mass and deterioration of the microarchitecture, resulting in an increase in fragility and an increased susceptibility to fracture (Raisz, 2005). Currently, 50% of women and 20% of men over the age of fifty will have a fragility fracture, and there are an estimated 158 million people over the age of 50 at high risk of osteoporotic fracture worldwide, with this predicted to double by 2040 (Odén et al., 2015). In 2010, the financial burden of treating osteoporotic fractures in the UK and Germany was estimated to be €5.5 billion and €9.1 billion, respectively (Ström et al., 2011). However, the demographics of western countries are changing with an increasing proportion of the population exceeding 50 years of age, thus further increasing the incidence and cost each year (Egermann et al., 2005). For example, the financial burden in Canada was estimated to be $2.3 billion in 2008, but this was revised to $4.6 billion in 2016 (Hopkins et al., 2016).

### The need for *in vitro* models

In 2015, 2.08 million experimental procedures were performed on animals in the United Kingdom. 1.1 million were for basic research purposes, with 25,381 of these within the field of musculoskeletal research. 89% of these studies were conducted on mice and rats (Home Office UK, 2015). The popularity of rodent models for bone disorders arises from a relatively minimal public opposition to their use, as well as low cost and ease of housing in comparison to other, larger animal alternatives (Bonucci and Ballanti, 2013). Furthermore, their size makes them amenable to non-invasive, high resolution *in vivo* imaging techniques such as micro-CT (van der Linden et al., 2006; Dall'Ara et al., 2016) and the application of mechanical loading *in vivo* (Gross et al., 2002). However, despite becoming a fundamental component of pre-clinical research, mouse physiology does not accurately represent the human condition, with many aspects of human anatomy not well represented in a murine model. This is demonstrated by the poor translation of pre-clinical efficacy in animal models to human clinical trials and the vast majority of promising discoveries failing to enter routine clinical use (Burkhardt and Zlotnik, 2013; Mcgonigle and Ruggeri, 2013; Benam et al., 2015; Malfait and Little, 2015).

Although *in vivo* models are viewed as the gold standard for studying diseases and testing new therapies, their use should align with the philosophy of the 3Rs – reducing, refining and replacing animal testing (Peric et al., 2015). In addition to these principles, in September 2010 the EU Directive “Directive 2010/63/EU—Legislation for the protection of animals used for scientific purposes” was adopted for research in EU nations. This supports the principles of the 3Rs, widening their scope and laying down standards for housing and care of animals. Furthermore, it aims establishes a Union reference laboratory for the validation of alternatives to animal models in order to promote their development, validation and implementation (European Union, 2010). These limitations of *in vivo* models give rise to the development of *in vitro* alternatives. However, the clinical relevance of these systems should be interpreted with caution as they lack the complexity of *in vivo* physiology. Despite this, if aspects of preclinical testing can be replicated or improved on *in vitro* before proceeding to *in vivo* then the use of some animals can be reduced.

### Types of *in vitro* model

**Figure 4:**
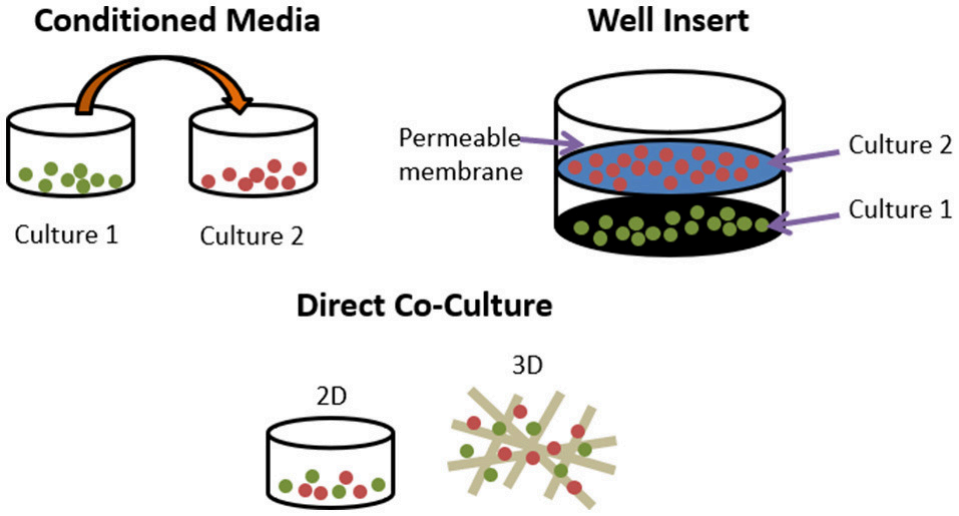
Different methods of co-culturing cells. Conditioned media transfers media used in one culture to another. Well inserts culture cells in the same well but only soluble factors can exchange between cell types. Direct co-cultures can be performed in 2D or 3D and permit membrane bound and soluble factors to exert influence.

Even the most minimal *in vitro* models of bone remodelling fundamentally require the co-culture of osteoblasts and osteoclasts. However, these can be performed either indirectly or directly. Indirect methods include conditioned media and the use of transwell inserts. The former takes media from one cell type and adds it to another, whereas the latter uses a permeable insert to provide two culture surfaces in the same well, allowing exchange of soluble factors but no cell-cell contact between the two types. Direct methods co-culture both cell types on the same surface, be it a planar, two-dimensional (2D) tissue culture well or a three-dimensional scaffold (Figure 4). This approach allows for immediate physical contact between the cells types, meaning that the effects of membrane-bound signalling molecules can be explored as well as soluble factors.

A recent review suggested that when testing bone implant and repair materials *in vitro*, overlooking bone remodelling is a key limitation of the approaches currently taken (Kohli et al., 2018). That paper makes a strong case for improving our ability to replicate this process for *in vitro* biomaterial testing; therefore, biomaterials testing is not discussed in this review. Rather, the current review summarises the key research that has been done thus far in creating an *in vitro* model of bone remodelling, focussing on fundamentals, 3D models of the process, and disease orientated-models.

We searched in PubMed for “*in vitro* AND osteoblast AND osteoclast AND (co-culture OR co culture) AND remodelling,” limiting results to relevant original research articles written in English. The resulting papers were divided into three categories: modulators of remodelling; which explores which factors can affect the remodelling process, remodelling models; which attempt to mimic the process *in vitro* as a tool for understanding physiology or drug/material testing, and disease-orientated models; that introduce additional cells or factors (e.g., cancer cells or inflammatory molecules) to the osteoblast-osteoclast co-culture to investigate their effects on remodelling. Common osteoclastic and osteoblastic cell types used are summarised in Tables 1, 2, and proteins, genes and molecules referred to throughout this review are listed in Table 3.

**Table 1:** Common osteoclastic lineage cell types used *in vitro*.

**Name**	**Abbreviation**	**Description**
Peripheral Blood Mononuclear Cell	PBMC	Mononuclear cells (predominantly lymphocytes and monocytes) isolated from peripheral blood or buffy coats, typically via density gradient centrifugation (Henriksen et al., 2012).
Monocyte	MNC	Monocytes (osteoclast precursors) can be further isolated from PBMCs, for example by negative selection using magnetic-activated cell sorting or adhesion-selection. Their purity can be confirmed by flow cytometry using antibodies against CD14 and CD45 (Domaschke et al., 2006; Anaraki et al., 2015).
Bone marrow derived precursors	–	Bone marrow consists of haematopoietic tissue and the supporting stroma. Non-adherent cells within the former can be isolated and differentiated into osteoclasts (Gori et al., 2000; Lindner et al., 2010).
RAW264.7 (ATCC® TIB-71™)	RAW264.7	Murine leukemic monocyte macrophage cell line that can undergo osteoclastic differentiation by RANKL exposure. A key advantage over other precursors is that they do not require co-stimulation with M-CSF (Collin-Osdoby et al., 2003; Zhang et al., 2012).
THP-1	THP-1	Human monocytic cell line derived from the blood of a boy with acute monocytic leukaemia (Tsuchiya et al., 1980).

**Table 2:** Common osteoblastic lineage cell types used *in vitro*.

**Name**	**Abbreviation**	**Description**
Primary Osteoblast	-	Osteoblast-like cells extracted from primary bone. Human osteoblasts are typically from trabecular bone fragments (Tanaka et al., 2000; Xie et al., 2012), murine osteoblasts from calvaria (Tortelli et al., 2009; Vinik et al., 2015).
Mesenchymal stem cell	MSC	Bone marrow consists of haematopoietic tissue and the supporting stroma. A subpopulation of adherent stromal cells within the latter have multipotent differentiation capacity (e.g., osteoblasts, chondrocytes, adipocytes) and are commonly referred to as mesenchymal stem cells. Similar cells have also been derived from other tissues (e.g., adipose and umbilical cord) (Lindner et al., 2010).
Immortalised osteoblast precursors from human bone marrow stroma	hMS(2-15)	Osteoblast precursor cell line developed from human bone marrow stromal fraction (Hicok et al., 1998).
ST-2	ST-2	Clone of murine stromal cells isolated from BC8 mice that develop an osteoblastic phenotype when cultured with ascorbic acid (Otsuka et al., 1999).
MC3T3-E1	MC3T3-E1	Spontaneously immortalised clonal osteoblast precursor cell line generated using the 3T3 passaging protocol from the calvaria of newborn C57BL/6 mice by Kodama et al. (1981).
Human periodontal ligament cells (between alveolar bone and the tooth root)	PDL	Osteoprogenitor cells of periodontal ligament connective tissue (Basdra and Komposch, 1997; Chou et al., 2002).
MLO-Y4	MLO-Y4	Osteocyte cell line cloned from cells isolated from murine long bones (Kato et al., 1997).

**Table 3:** Common factors analysed during *in vitro* bone cultures.

**Name**	**Abbreviation**	**Description**
Receptor activator of nuclear factor κβ	RANK	Receptor for RANKL expressed on osteoclast-lineage cells (Boyce and Xing, 2007).
Receptor activator of nuclear factor κβ ligand	RANKL	Member of tumour necrosis factor cytokine family. Ligand for RANK receptor predominantly produced by osteoblast-lineage cells, but also by stromal and T cells (Boyce and Xing, 2007).
Osteoprotegerin	OPG	Decoy receptor that prevents RANK activation by binding with RANKL (Boyce and Xing, 2007).
Macrophage colony stimulating factor	M-CSF	Cytokine that influences differentiation and survival of haematopoietic precursors, produced by osteoblasts and stromal cells (Hodge et al., 2007).
Alkaline phosphatase	ALP	Enzyme secreted from osteoblasts which promotes hydroxyapatite crystal formation within the bone matrix. Considered a highly specific marker of bone-forming osteoblasts (Orimo, 2010).
Collagen type 1	COL-1	Protein that constitutes ~90% of the organic phase of bone (Farbod and Nejadnik, 2013).
Runt-related transcription factor 2	RUNX2	Key transcription factor associated with osteoblast differentiation (Komori, 2010).
Osterix	OSX	Transcription factor also known as Sp7 required for bone formation, works downstream of RUNX2 (Nakashima et al., 2002).
Osteopontin	OPN	OPN is an extracellular matrix glycoprotein. During remodelling, it anchors osteoclasts to the bone matrix (Reinholt et al., 1990).
Integrin binding sialoprotein/Bone sialoprotein-2	IBSP/BSP-II	Human variant of BSP, significant component of bone extracellular matrix (Fisher et al., 1990).
Tartrate-resistant acid phosphatase	TRAP	Enzyme secreted by osteoclasts. Activity strongly correlates with bone resorption and TRAP knockout mice develop osteopetrosis (Hayman et al., 1996).
Cathepsin K	CTSK	Osteoclastic cysteine protease that catabolises bone by breaking down collagen (Sprangers and Everts, 2017).
Matrix metalloproteinase-9	MMP-9	Osteoclastic enzyme that degrades extracellular matrix components such as collagen and gelatin (denatured collagen) (Sprangers and Everts, 2017).
Osteoclast associated receptor	OSCAR	An IgG-like receptor that is an important osteoimmunological mediator and acts as a co-stimulatory receptor for osteoclast differentiation (Nemeth et al., 2011).
Parathyroid hormone	PTH	A hormone that can indirectly stimulate osteoclastogenesis by action on osteoblasts. Depending on concentration and frequency of application, it can have a catabolic or anabolic effect (Borba and Mañas, 2010).
1α,25(OH)_2_D_3_	Vitamin D_3_	1α,25-dihydroxyvitamin D3 is the active form of vitamin D3. It has been shown to stimulate RANKL expression in osteoblasts and osteocytes (Kitazawa and Kitazawa, 2002; Shevde et al., 2002; You et al., 2008).

## Modulators of remodelling

Since the 1980s it has been known that osteoblast-lineage cells release factors that stimulate osteoclastic resorption, and *in vitro* experiments were essential in their elucidation. For example, the discovery of interleukin-6 and M-CSF, as well as the RANKL/RANK/OPG axis that mediates osteoclast formation and function (McSheehy and Chambers, 1986; Ishimi et al., 1990; Greenfield et al., 1992; Jimi et al., 1996; Pacifici, 1996; Feng and McDonald, 2011). M-CSF and RANKL can be produced as either membrane bound (mM-CSF/mRANKL) and/or secreted, soluble forms (sM-CSF/sRANKL). Factors that can modulate the expression of M-CSF, RANKL and OPG can direct whether bone remodelling has a negative, neutral or positive balance, controlling whether bone tissue is lost or gained.

Throughout this review a range of culture media is used depending on the cell type(s) being grown. Due to the range of studies discussed, the exact formulation cannot be detailed each time. However, osteogenic medium for the growth and differentiation of osteoblasts typically refers to a culture medium supplemented with dexamethasone, β-glycerolphosphate (βGP) and ascorbic acid, although the concentration of each varies between research groups. A comprehensive review of their roles is given by Langenbach and Handschel; but to summarise briefly, dexamethasone is a glucocorticoid that upregulates osteogenic differentiation, βGP acts as a phosphate source for the production of hydroxyapatite and ascorbic acid is a co-factor for enzymes involved in collagen synthesis (Langenbach and Handschel, 2013). Although these supplements are added to permit osteoblast differentiation and function, they have each been associated with enhanced osteoclast formation (Kaji et al., 1997; Takuma et al., 2003; Le Nihouannen et al., 2010; Noh and Yim, 2011). Osteoclastogenic medium typically refers to culture medium supplemented with RANKL and M-CSF. Where other, non-standard factors are added to the medium, e.g., vitamin D or parathyroid hormone, these are mentioned in the text.

### The RANKL:OPG ratio changes with progression through the osteoblast lineage

Although RANKL and OPG are produced by cells of the osteoblastic lineage, their expression varies depending on the progression of their differentiation from precursor to osteocyte. Culture of hMS(2-15) immortalised osteoblast precursors in osteogenic medium over 21 days sees an increase in ALP activity and mineralised matrix production over time. During this period, RANKL mRNA levels reduce and OPG mRNA levels are elevated in comparison to cultures in basal media, reducing the RANKL:OPG ratio. The presence of undifferentiated hMS(2-15) that have the greatest RANKL:OPG ratio results in the differentiation of murine bone marrow-derived osteoclast precursors into TRAP positive multinucleated osteoclasts in co-culture, whilst differentiated cells did not induce differentiation unless exogenous sRANKL was added (Gori et al., 2000).

However, the ability to generate osteoclasts does not necessarily diminish as osteoblastic cells continue to differentiate. Osteoblasts that terminally differentiate into osteocytes and become embedded within the mineralised matrix of bone also have the ability to modulate bone remodelling. MLO-Y4 osteocyte-like cells can induce the formation and activation of osteoclasts capable of resorbing dentine in direct co-culture with murine spleen or marrow cells without the addition of any exogenous factors, although supplementation with Vitamin D_3_ enhances their production. However, in an indirect co-culture where conditioned media was taken from an MLO-Y4 monoculture and added to an osteoclast precursor monoculture, osteoclasts were not generated despite sM-CSF being present. This indicates that M-CSF alone cannot induce osteoclastogenesis, and that MLO-Y4 must stimulate osteoclast formation through the mRANKL detected on their surface and dendritic processes, meaning that direct cell contact is required in this culture system (Zhao et al., 2002).

Mechanical forces sensed and transduced at the cellular level can also affect the RANKL:OPG ratio. This mechanotransduction can occur through either fluid shear stress (FSS) via the primary cilium, glycocalyx or ion channels, or matrix strain via integrins (Wittkowske et al., 2016). It is thought that osteocytes inhibit bone resorption in areas of high mechanical loading by downregulating RANKL and upregulating OPG in response to changes in fluid flow that occur within the tissue. Kulkarni, et al., applied pulsatile fluid flow (PFF) to MLO-Y4, finding that this inhibited their ability to induce osteoclastogenesis in murine bone marrow cells by decreasing the RANKL:OPG ratio (Kulkarni et al., 2010). Kim, et al., also investigated the effect of fluid flow on RANKL and OPG expression by applying oscillating fluid flow (OFF) to ST-2 stromal cells before initiating a direct co-culture with RAW264.7 monocyte macrophages. OFF reduced the RANKL:OPG ratio by lowering RANKL and stimulating OPG mRNA expression, resulting in fewer osteoclasts being formed in comparison to a static control (Kim et al., 2006).

Direct matrix strain can also affect the RANKL:OPG ratio. Using a transwell co-culture system where RAW264.7 were cultured on a membrane above mechanically strained MC3T3-E1 osteoblast precursors, Zhang et al. found that loading of the osteoblasts resulted in higher ALP activity and lowered osteoclast activity, as demonstrated by a decline in TRAP activity, resorption, CTSK and MMP-9 expression, in comparison to static controls. This was due to a lower RANKL:OPG ratio by reduced OPG expression (Zhang et al., 2013). Unlike with MLO-Y4 co-cultures, in this culture system, direct contact between osteoblast- and osteoclast-lineage cells was not necessary to induce osteoclastogenesis. This highlights how although RANKL can exist in both membrane-bound and soluble isoforms, both types are not necessarily present in sufficient concentrations to induce osteoclast differentiation in all osteoblast sources and culture conditions.

To investigate how osteocytes may regulate osteoclast activity, Gu et al. cultured primary rat osteoclasts on rat calvaria slices that had been stripped of the periosteum and endosteum to leave predominantly osteocytes in the samples. These were either cultured to maintain living cells or devitalised using water and sonication or freeze-thawing. Cultures on devitalised bone produced significantly more and deeper resorption pits in comparison to living bone, and conditioned media from living bone samples inhibited osteoclast resorption, indicating the live osteocytes were preventing resorption. Inducing osteocyte apoptosis via glucocorticoid application prior to osteoclast culture greater resorption in comparison to untreated calvaria (Gu et al., 2005). The alteration of this ratio over time may help co-ordinate the osteoblasts and osteoclasts during the remodelling cycle.

When considered in the context of remodelling and bone homeostasis *in vivo*, the need for the osteoclastogenic potential of osteoblast-lineage cells to change as they progress from mesenchymal stem cells (MSC) to osteoblast to osteocyte is clear. During the initial stages of remodelling when osteoblast and osteoclast precursor cells are being recruited from the circulatory system, monocytes need to differentiate into mature osteoclasts so that the cutting cone of the BMU can develop and resorb the bone tissue; therefore, a RANKL:OPG ratio that promotes osteoclast formation is necessary. However, once osteoblasts are synthesising new osteoid, osteoclastogenesis is not desirable as it could result in the resorption of the newly formed bone tissue; hence the reduction in the RANKL:OPG ratio. Once terminally differentiated into osteocytes, it is important that bone strength is preserved. Therefore, it is predicted that in loaded regions of bones osteocytes have a low RANKL:OPG ratio to minimise resorption, whereas in unloaded or damaged areas of bone, the RANKL:OPG of the osteocytes is higher to promote bone remodelling.

### Other proteins and hormones that can modulate the RANKL:OPG ratio

There are a wide range of factors which can modulate the RANKL:OPG ratio and therefore osteoclastogenesis and bone remodelling. Retinoids are important for normal bone growth and development. Geranylgeranoic acid (GGA), a synthetic acyclic retinoid, can promote a positive bone balance through a dual action of stimulating osteoblast differentiation and inhibition of osteoclast formation, as demonstrated by Wang, et al., who found it suppressed MC3T3-E1 proliferation whilst increasing ALP activity, reduced osteoclast formation in co-cultures of murine bone marrow cells and osteoblasts, and upregulated OPG mRNA expression in ST-2 cells after it had been chemically supressed. Furthermore, it inhibited osteoclast formation in sRANKL and sM-CSF treated bone marrow cultures, indicating it can act on both osteoblasts and osteoclasts (Wang et al., 2002).

Hydrolysed collagens have also been shown to promote a positive bone balance *in vitro*, with 2 kDa hydrolysed collagen molecules increasing ALP activity and decreasing resorption in co-cultures of primary murine osteoblasts and bone marrow cells (Guillerminet et al., 2010). The presence of omentin-1, an adipokine also known as intelectin-1, reduces the number of TRAP positive, multinucleated osteoclasts in co-cultures of human osteoblasts and monocytes (MNCs) and well as MC3T3-E1 and RAW264.7 through stimulation of OPG and inhibition of RANKL protein expression (Xie et al., 2012). Lactoferrin is a glycoprotein that has an anabolic effect on bone by promoting osteoblast proliferation and differentiation as well as decreasing osteoclast formation (Lorget et al., 2002; Zhang et al., 2014). By coupling it to hydroxyapatite nanocrystals, Montesi et al. showed that these two compounds can synergistically act as a bone anabolic by increasing OSX and IBSP mRNA expression in osteoblasts, reducing osteoclast formation, and downregulating OSCAR and CTSK mRNA expression in co-cultures of MC3T3-E1 and RAW264.7 (Montesi et al., 2015).

Galectins are glycan-binding proteins that can link ECM components and cell-surface receptors. Vinik et al. investigated how galectin-8 (GAL-8) can regulate RANKL production by co-culturing murine osteoblasts with murine bone marrow cells and treating with GAL-8. They found that GAL-8 can bind to the LRP1, MRC2, and uPAR receptors and stimulate a six-fold increase in RANKL mRNA expression and a 2.5-fold increase in sRANKL production whilst reducing OPG expression by 30%, resulting in a 15-fold increase in TRAP positive, multinucleated osteoclast generation. Furthermore, osteocytes extracted from the same calvaria treated with GAL-8 also had higher RANKL expression (Vinik et al., 2015).

Glycosaminoglycans (GAGs) are found in the ECM of bone and have been used in biomaterial applications to stimulate osteogenesis. Salbach-Hirsch et al. used GAGs and sulphated-GAGs (sGAGs) to create artificial ECMs for culturing RAW264.7 in the conditioned media of murine osteoblasts generated from MSCs. They found that sGAG matrices significantly increased ALP, osteocalcin and OPG expression as well as mineralized matrix deposition. However, supernatants could not induce osteoclast formation due to the absence of sRANKL and the high level of OPG. Addition of exogenous sRANKL permitted osteoclast formation, but the lowest levels of differentiation and resorption were seen in the sGAG groups due to the greater OPG production, indicating that GAGs can have an anabolic effect on bone through action on the osteoblasts (Salbach-Hirsch et al., 2014).

**Figure 5:**
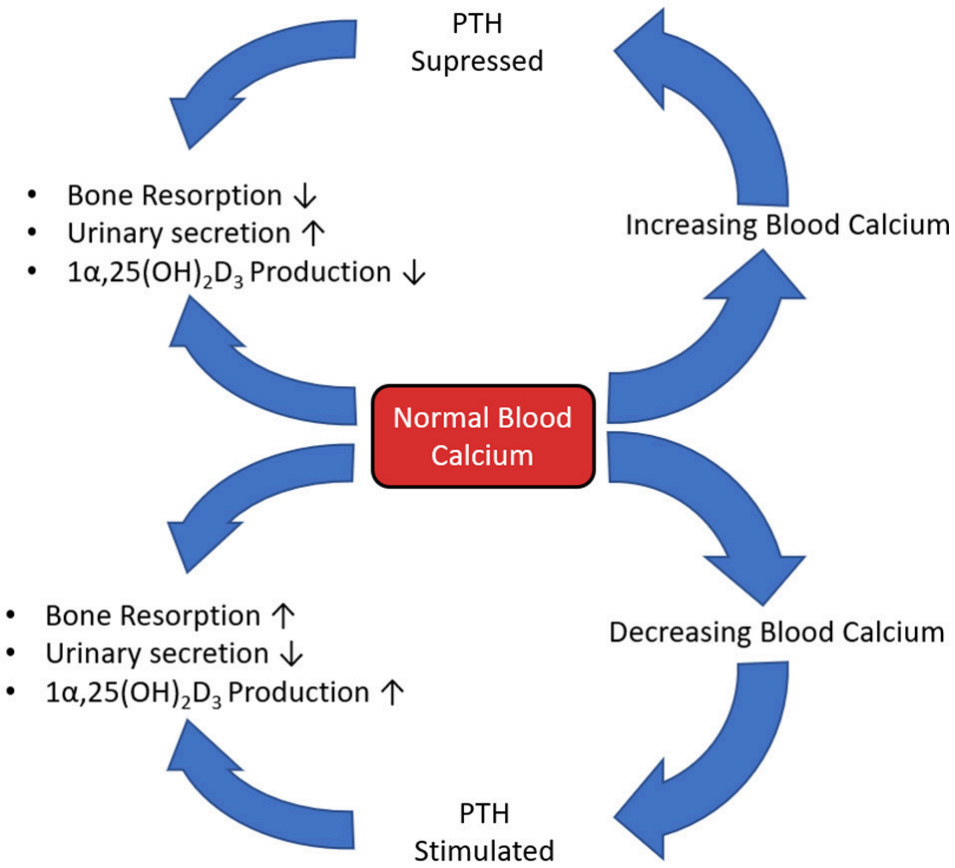
The calcium, PTH, Vitamin D_3_ homeostatic feedback loop.

Parathyroid hormone (PTH) plays a major role in calcium and phosphate homeostasis (Figure 5). When this feedback loop is disrupted, for example when PTH is continuously produced in hyperthyroidism, there is a catabolic effect on bone. This is partly the result of the inhibition of expression and synthesis of ECM proteins, such as collagen I, osteocalcin, and ALP. However, despite this reduced matrix formation, PTH mediated catabolism of bone is not primarily due to diminished osteoblast function, but elevated osteoclast function. The prevailing scientific opinion is that only osteoblast-lineage cells possess a PTH receptor, with only a small number of researchers reporting that they are also present on osteoclast-lineage cells. Therefore, this raised osteoclast activity is not through direct action of PTH, but rather an indirect response to altered osteoblast function as continuous PTH raises RANKL and lowers OPG expression, increasing the RANKL:OPG ratio (Dempster et al., 2005; O'Brien et al., 2008; Silva and Bilezikian, 2015; Liu et al., 2016).

**Figure 6:**
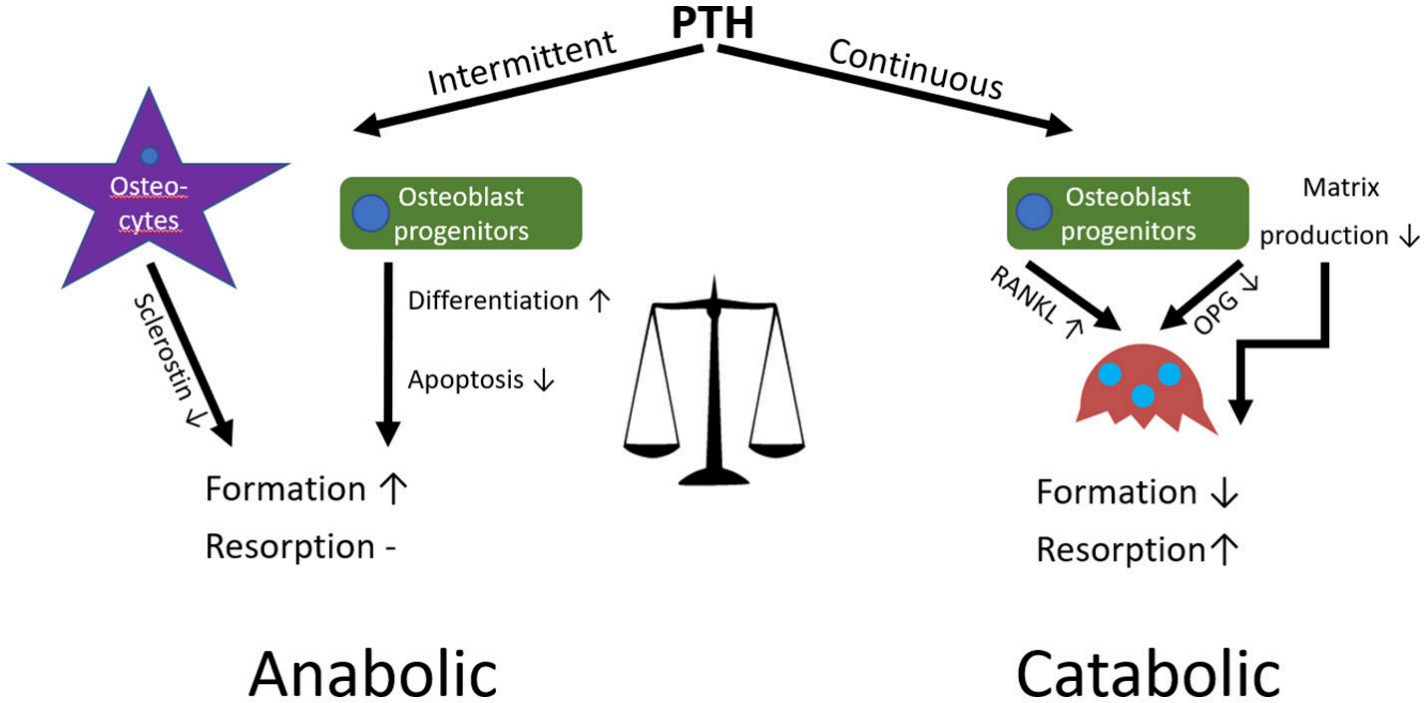
The anabolic or catabolic effects of PTH on bone depends on application modality.

In contrast to the loss of bone observed when PTH is continuously applied, intermittent application of exogenous PTH, for example the application of teriparatide in the treatment of osteoporosis, can have an anabolic effect on bone by increasing formation in both cancellous and cortical regions (Figure 6). This is because intermittent administration stimulates bone formation by promoting bone-lining cell activation and MSC differentiation to osteoblasts, inhibiting sclerostin in osteocytes, and increasing osteoblast lifespan by inhibiting apoptosis (Lindsay et al., 1997; Kousteni and Bilezikian, 2008; Borba and Mañas, 2010; Silva and Bilezikian, 2015). Although this can seem paradoxical, the anabolic effect is due to the immediate response of osteoblasts in contrast to the delayed secondary response of osteoclasts after administration. This differential gives rise to an “anabolic window,” where there is an initial stimulation of formation without resorption (bone modelling) followed by a later increase in overall bone turnover (bone remodelling). After each PTH cycle there is a net increase of high quality bone similar to that of younger individuals, predominantly at regions subjected to mechanical stress; hence its potency as an osteoporosis treatment (Bodenner et al., 2007; Silva and Bilezikian, 2015).

Osteoclastogenesis and bone resorption can also be affected by modulating the expression of or by knocking out (KO) proteins involved in the signalling pathways downstream from cell surface receptors. This can be done directly in osteoclasts or indirectly by affecting the RANKL:OPG ratio in osteoblasts. Although altering these signalling cascades is a potent method of modifying remodelling, as these pathways are not specific to the process being examined its use as a potential therapeutic should be treated with caution. Below are two examples of how knocking out proteins in these signalling pathways can affect osteoclastogenesis. As the intricacies of how these pathways function is outside the scope of this review, only a brief explanation of their function is given here.

β-arrestin2 is an adaptor/scaffold protein that can regulate intracellular signalling initiated by the PTH-receptor, when β-arrestin2 KO vs. wild-type (WT) bone marrow cultures are compared, enhanced osteoclastogenesis is seen in the KO phenotype due to a higher RANKL:OPG ratio (Pierroz et al., 2009). Lossdörfer et al. co cultured periodontal ligament (PDL) cells with RAW264.7 in the presence of intermittent PTH, finding that the response depends on the maturity of the PDL cells. When applied to co-cultures containing mature, confluent PDL cells, an upregulation of TRAP and CTSK expression and an increase in the RANKL:OPG ratio and resorptive activity was observed; however, in less mature, pre-confluent cells the opposite was seen with downregulation of resorptive genes, and a decrease in the ratio and amount of resorption. Similar results were seen when treating RAW264.7 with conditioned media from the PDL cells, indicating the PDL cells can produce sRANKL and their response to PTH is dependent on cell maturity (Lossdörfer et al., 2011).

Downstream of tyrosine kinase (DOK) 3 protein is an adapter protein that limits tyrosine kinase-mediated signalling downstream of cell surface receptors such as *c-fms* on osteoclasts (Mashima et al., 2009). Cai et al. found that DOK3 KO mice are osteoporotic due to a higher number of TRAP positive osteoclasts. *In vitro*, osteoclasts differentiated from KO bone marrow had heightened sensitivity to RANKL resulting in amplified osteoclastogenesis, larger osteoclasts with more nuclei and enhanced resorptive capability. Osteoblasts derived from DOK3 KO mice produce less mineralised matrix and reduced expression of RUNX2, OSX, COL-1, and ALP mRNA, as well as reduced RANKL and increased OPG expression in comparison to WT. In direct co-culture, WT osteoblasts induced more osteoclasts from KO precursors than WT precursors and KO osteoblasts induced fewer osteoclasts from KO and WT precursors that WT osteoblasts, indicating that DOK3 promotes osteoblastogenesis and impedes osteoclastogenesis (Cai et al., 2017).

### The influence of culture media

Serum, typically foetal bovine serum, is added to culture media as it contains a variety of proteins that help cells grow, divide and survive. However, its composition is not fully defined and therefore varies between batches (Baker, 2016). This can give rise to inconsistencies in results due to the influence of unknown factors in the medium. Atkins et al. attempted to develop a human-derived co-culture in a defined serum-free medium. They found that in serum-replete medium, ST-2 osteoblasts supported the formation of osteoclasts from human peripheral blood mononuclear cells (PBMCs) in co-culture. However, when the ST-2 cells were replaced with human osteoblasts, osteoclast formation ceased. When repeated in a defined, serum-free medium, co-culture of human osteoblasts with osteoclast precursors resulted in functional osteoclast formation, indicating some factor may have been present in the serum that inhibited osteoclastogenesis (Atkins et al., 2005).

The concentration of calcium and phosphate in the media can also affect osteoclastogenesis. Co-culture of MC3T3-E1 murine pre-osteoblasts with murine bone marrow cells on segments of bovine tooth-roots with calcium concentrations ranging from 0 to 2.5 mM found increased TRAP staining and resorption with lower calcium (Shirai et al., 1999). The same group found that as MC3T3-E1 ALP activity and therefore extracellular inorganic phosphate increases over time there is diminished osteoclast formation, and that increasing concentrations of β-glycerophosphate (βGP) and exogenous ALP in the media has a similar effect (Takeyama et al., 2001).

### Environmental factors

In addition to chemical stimulus, environmental factors can also mediate osteoclastogenesis. Dandajena et al. indirectly co-cultured human osteoblasts with human PBMCs using well inserts in either normoxic (21% O_2_) or hypoxic (2.5% O_2_) conditions, finding that low oxygen significantly upregulated RANKL production in comparison to normoxia, and that TRAP positive, resorptive osteoclasts only formed in hypoxic co-cultures (Dandajena et al., 2012).

### The effect of osteoclasts on osteoblasts

Although the relationship between osteoblasts and osteoclasts is perhaps often considered to be one-sided, with the former producing factors that modulate the latter's behaviour, there is increasing evidence that osteoclasts can also affect osteoblasts. This has seen a shift in thinking from the assumption that the osteoclast is a single-function cell type that destroys the tissue it resides in to understanding that it has additional regulatory roles in bone remodelling (Cappariello et al., 2014).

Garimella et al. co-cultured primary murine calvarial osteoblasts with murine bone marrow cells to generate TRAP positive, multinucleated osteoclasts. Using *in situ* hybridisation and immunohistochemistry they were able to detect bone morphogenetic protein (BMP)-2, -4, and -6 mRNA and protein, respectively, within the osteoclasts. RT-PCR confirmed that the osteoclasts could synthesise mRNAs for BMP-2, -4, -6, and -7, with BMP-6 having the highest expression (Garimella et al., 2008). Osteoclastic BMPs are possibly involved in the initiation of the anabolic phase of remodelling, recruiting, and activating osteoprogenitor cells (Pederson et al., 2008). Further evidence for the influence of osteoclasts on osteoblasts comes from Luo et al. who also co-cultured primary murine calvarial osteoblasts with murine bone marrow cells, albeit in a transwell configuration resulting in no direct osteoblast-osteoclast contact. Their findings demonstrated greater ALP staining and activity in osteoblasts co-cultured with osteoclasts in comparison to monocultures. Furthermore, the osteogenesis related genes RUNX2, ALP, and COL-1 mRNA expression were all upregulated in the co-culture, indicating the anabolic role osteoclasts can have on osteoblasts (Luo et al., 2016).

## Remodelling models

Much of the work done on the fundamentals of bone remodelling *in vitro* has been performed using either (1) conditioned media experiments, (2) indirect co-culture using well inserts that keep the osteoblasts and osteoclasts separate, or (3) in direct co-culture, where osteoclasts are plated onto a monolayer of osteoblasts grown on tissue culture plastic. Whilst these studies have revealed a plethora of factors and molecules involved in remodelling, they fail to replicate the 3D architecture of native bone tissue. This results in differences in cell morphology, polarity and receptor expression, as well as a lack of diffusion gradients and unrepresentative substrate stiffness that in combination fail to represent the *in vivo* condition (Baker and Chen, 2012). Therefore, to produce a viable model of remodelling *in vitro*, the co-culture should be performed in three dimensions. The development of the field of bone tissue engineering has resulted in a multitude of polymer, ceramic and metal scaffolds being produced that support the formation of mineralised ECM (Bose et al., 2012; Owen et al., 2015). Those studies primarily focus on the action of osteoblasts but such scaffolds can be adapted to create *in vitro* models of remodelling.

Nakagawa et al. appear to be the first to attempt to co-culture osteoblasts and osteoclasts in a scaffold *in vitro*. Collagen-coated porous PLGA scaffolds were precultured with porcine osteoblasts for 2 weeks before the addition of porcine osteoclast precursors and a further 2 weeks of culture in a rotational bioreactor. At the end of the culture, mature osteoclasts with ruffled borders and actin rings were visible on top of the mineralised surface synthesised by the osteoblasts, showing for the first time that studying remodelling *in vitro* is a realistic ambition (Nakagawa et al., 2004).

Domaschke et al. were the first to demonstrate remodelling *in vitro* and recognise its potential to reduce the need for animal studies. Human MNCs were cultured on mineralised collagen tapes containing hydroxyapatite for 24 h before the addition of ST-2 and cultured in media supplemented with exogenous RANKL and M-CSF. Osteoclasts generated were able to resorb the scaffold-substrate and osteoblasts were able to deposit new mineralised matrix, a key element that distinguishes remodelling from resorption (Domaschke et al., 2006). Further work by Bernhardt et al. utilised the same substrate but replaced the murine ST-2 cells with primary human MSCs. However, here osteoblasts were seeded on one set of scaffolds and monocytes on another and kept separate from each other using well inserts. Osteoblasts proliferated at a faster rate and ALP mRNA expression was higher in co-culture than monoculture. TRAP activity was lower on scaffolds and osteoclasts were smaller in size in the co-culture in comparison to monocyte monocultures. However, there was no difference in CTSK and TRAP mRNA expression. Interestingly, across multiple donors MSCs underwent less adipogenic differentiation in co-culture than in monoculture, as evidenced by fatty acid binding protein 4 expression and Oil Red O staining (Bernhardt et al., 2010). This indicates that without direct contact the presence of osteoblasts can have an inhibitory effect on osteoclasts, perhaps via the higher concentration of extracellular phosphate in the media due to the presence of ALP as seen by Takeyama et al. (2001). Furthermore, the presence of osteoclasts in indirect co-culture upregulated the proliferation and ALP activity of osteoblasts and inhibited adipogenesis, which agrees with the finding of Luo et al. (2016).

Following the initial work of Domaschke, Tortelli et al. developed an *in vitro* remodelling model using primary murine cells. They used Skelite®, a commercial bone graft substitute, formed into discs to create a porous ceramic substrate and compared it to TCP using co-cultures that were seeded in a 1:1 ratio of murine osteoblasts and osteoclast precursors from murine bone marrow. These cultures were maintained for 30 or 60 days, 2–4 times longer than Domaschke et al. without exogenous RANKL or M-CSF before analysis. Mature, TRAP positive, multinucleated osteoclasts and deposited, mineralised ECM were detectable after 60 days in 2D and 3D, with a more organised bone-like matrix deposited in 3D. RUNX2, OSX, and osteocalcin expression were analysed as markers of early, middle and late osteoblast maturation and CTSK and TRAP expression as markers of osteoclast differentiation. 3D cultures reached a maximal expression of RUNX2 and OSX within 10 days, whereas 2D cultures took 40 days, and osteocalcin expression was 19-fold higher in 3D by day 40, indicating osteogenesis started immediately in 3D but was delayed in 2D, and that osteoblasts fully differentiated in 3D. CTSK, TRAP, and RANKL expression were significantly higher in 2D than 3D, whereas OPG expression was lower (Tortelli et al., 2009). These results combine to imply that osteoblasts in the earlier stages of osteoblastic differentiation have higher osteoclastogenic potential that more mature osteoblasts due to a greater RANKL:OPG ratio, and that 2D culture retains osteoblasts in an earlier phenotype due to a lack of physical stimuli, which agrees with the findings on Gori et al. (2000).

Although bone turnover at various time points can be analysed by histology, PCR and enzyme activity, it is difficult to determine exactly how a scaffold has been resorbed and remodelled by the cells and impossible to see how bone tissue volume on the same scaffold changes over time due to the destructive nature of these techniques. Ruggiu et al. repeated the same co-culture as Tortelli et al. on the same substrate but in addition to histological techniques, examined the scaffold before and after culture by x-ray computed microtomography (mCT) using a synchrotron to enable image registration. In comparison to murine OB monocultures, co-cultures formed a more organised bone tissue with clear segregation between mineralised ECM and non-mineralised osteoid. Using mCT, more extensive mineralised and non-mineralised matrix deposition was seen in co-cultures, as well as scaffold degradation due to osteoclast activity which was not visible in murine OB monocultures (Ruggiu et al., 2014).

**Figure 7:**
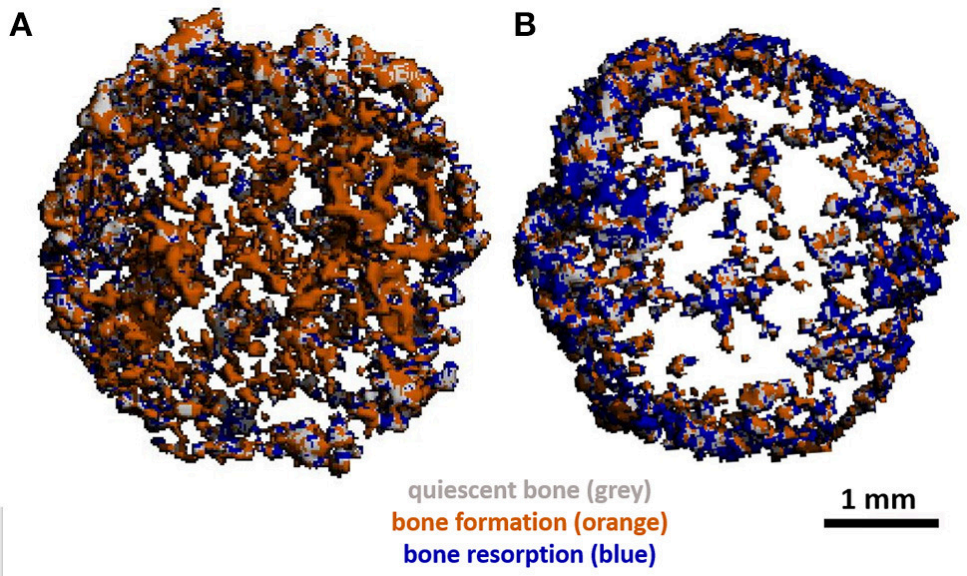
**(A)** Day 21 and **(B)** day 35 mCT scans of human osteoblast and osteoclast co-cultures registered to the original scaffold. Newly formed bone is coloured orange, resorbed areas are blue, constant/quiescent areas are grey. Adapted from (Rubert et al., 2017) under The Creative Commons Attribution–ShareAlike License (CC-BY-SA).

The use of mCT as a non-invasive imaging technique to monitor remodelling *in vitro* has also been utilised by Rubert et al. who co-cultured human MSCs and MNCs on previously mineralised and decellularised bone-like tissues in a spinner flask for up to 35 days on media supplemented with RANKL and M-CSF. By evaluating dynamic morphometric parameters using sequential mCT scans, co-cultures had a significantly decreased mineralising surface and almost 200% increase in bone resorption rate in comparison to human MSC mono-cultures. By registering images over time, regions of clear bone resorption and formation could be seen in the co-culture (Figure 7) (Rubert et al., 2017).

In addition to improving our understanding of the remodelling process, *in vitro* systems can also inform us how potential bone tissue engineering scaffolds will degrade and be remodelled *in vivo* prior to animal testing. Jones et al. co-cultured of MC3T3-E1 and primary murine osteoclast precursors on vapour or methanol stabilised silk fibroin, chitosan and PLLA films to determine their suitability for bone tissue engineering. Cultures were seeded at a 1:100 (OB:OC) ratio and maintained in media containing exogenous RANKL for 10 days. Silk and chitosan films supported the formation of superior numbers of TRAP positive osteoclasts in comparison to PLLA, and by comparing surface roughness using atomic force microscopy (AFM), the most resorption occurred on vapour stabilised silk films, indicating their potential for remodelling studies and use in bone tissue engineering (Jones et al., 2009).

Hayden et al. also utilised silk-based substrates in multiple studies attempting to develop an *in vitro* bone remodelling model. Initially they used lentiviral transduction to tether ligands known to alter bone metabolism: either PTH or glucose-dependent insulinotropic peptide (GIP), or a GFP control to human MSCs differentiated into osteoblasts. These were co-cultured with THP-1 monocytes in media with exogenous RANKL on silk films, porous silk sponges or TCP for up to 5 weeks before surface roughness and calcium deposition quantification was performed to analyse resorption and matrix deposition. Tethering of PTH increased mineral deposition in comparison to the GFP control in 2D co-culture only, possibly due to the feedback mechanism to the osteoblasts from the more active osteoclasts. Greater surface roughness was seen on silk films with PTH-tethered co-cultures due to the production of larger mineral deposits, whilst GIP-tethered co-cultures decreased roughness as GIP reduces osteoclasts activity and therefore osteoclastic stimulation of matrix deposition by osteoblasts. Similar surface roughness trends were seen in the 3D sponges, but due to their more complex architecture it is harder to quantify (Hayden et al., 2014a). Following this, Hayden et al. extended the duration of the co-culture with regular human MSCs and THP-1 and exogenous RANKL on the silk films to up to 32 weeks to characterise long term remodelling on the substrate. Films were characterised by SEM and mCT imaging prior to seeding. Mineralisation in co-culture in comparison to monoculture was continuous over the surface of the film, rather than in discrete patches. They also had higher surface roughness indicating more remodelling and an increase in volume as quantified by mCT (Hayden et al., 2014b). Finally, they looked to apply their *in vitro* model to a metabolic bone disease by investigating the effect of two bisphosphonates, a common therapeutic for patients with osteoporosis. Here the silk films were incorporated with hydroxyapatite and loaded with either clodronate or alendronate before co-culture of human MSCs and THP-1 with exogenous RANKL for up to 12 weeks. They identified concentrations of clodronate that could upregulate osteoblast ALP activity whilst diminishing osteoclast activity, a combination alendronate could not achieve (Hayden et al., 2014c).

**Figure 8:**
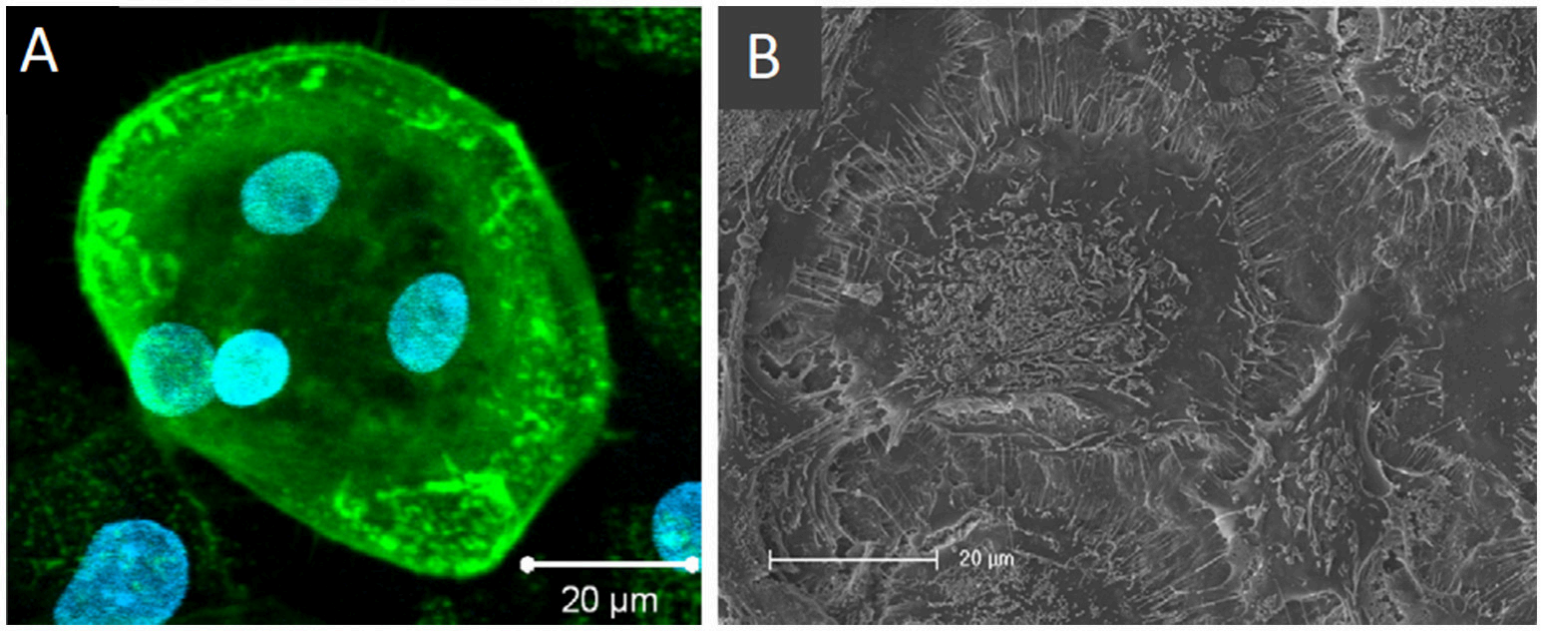
Osteoclasts within a 3D human osteoblast and osteoclast co-culture taken by **(A)** confocal laser scanning microscopy (cLSM) (actin—green, nuclei—blue) after 42 days and **(B)** SEM after 28 days. Osteoclast podosomes appear as dots within the cells by cLSM and thin filopodia are visible around the cell perimeter. Figure adapted from (Heinemann et al., 2011) under The Creative Commons Attribution–ShareAlike License (CC-BY-SA).

Heinemann et al. also developed an *in vitro* biomaterial testing system that utilised an all-human origin, direct contact co-culture that compared TCP with a silica-collagen-hydroxyapatite xerogel (Heinemann et al., 2011). Human MSCs were cultured for 13 days before the addition of human MNCs and further culture for up to 4 weeks without exogenous RANKL or M-CSF. Differentiation of MSCs into osteoblasts was confirmed by ALP activity. RANKL was synthesised as evidenced by RANKL mRNA expression and TRAP activity of the differentiated osteoclasts, which were also able to upregulate BSP-II gene expression in osteoblasts. In 3D, MSCs proliferated and differentiated, forming layers of cells that covered the entire sample which had spherical, multinucleated osteoclasts with actin rings embedded within (Figure 8).

**Figure 9:**
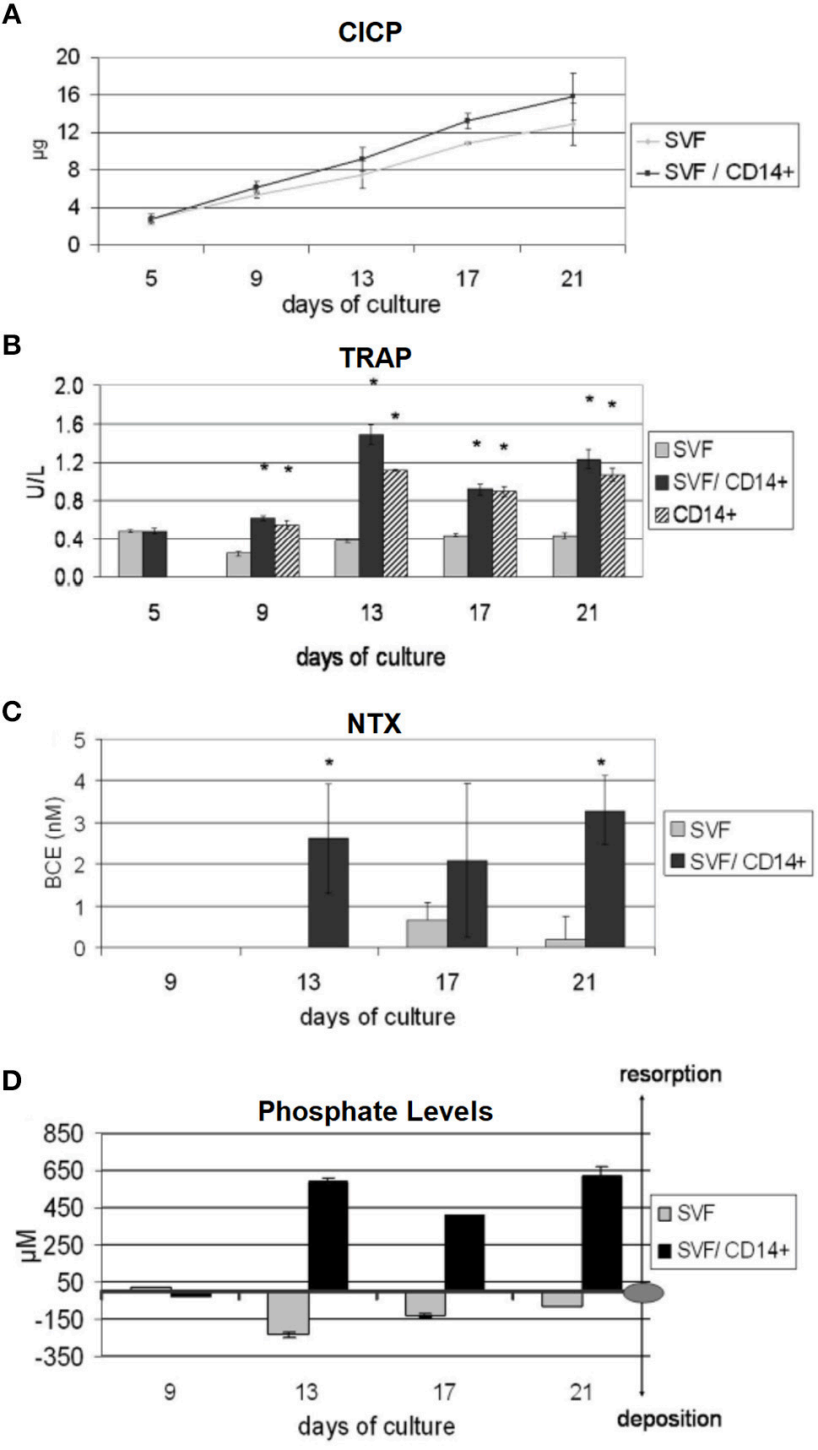
Non-invasive methods of assessing bone turnover *in vitro*. SVF cells can commit to osteoblastic and endothelial lineages. CD14+ cells differentiate to osteoclasts. Cell culture supernatants were analysed for **(A)** CICP, indicative of collagen synthesis, **(B)** TRAP, indicative of osteoclastic cells, **(C)** NTX, indicative of collagen resorption, **(D)** phopshate levels, where decreasing levels are indicative of mineralised matrix deposition and increasing levels are indicative of mineralised matrix resorption (^*^p < 0.05). Figure adapted from (Papadimitropoulos et al., 2011) under The Creative Commons Attribution-ShareAlike License (CC-BY-SA).

A further *in vitro* bone model that only used human-origin cells co-cultured in direct contact was developed by Papadimitropoulos et al. However, in contrast to Heinemann, they used human adipose tissue-derived stromal vascular fraction (SVF) cells that can commit to osteoblastic and endothelial lineages. These were co-cultured with human MNCs on porous hydroxyapatite/β-tricalcium phosphate ceramic scaffolds in a perfusion bioreactor, with the SVF cells cultured for 5 days before the addition of the monocytes and the culture maintained for 21 days in media supplemented with exogenous RANKL and M-CSF. After 21 days, osteoblastic, osteoclastic, and endothelial cells were identifiable in the culture, with TRAP positive osteoclasts adhered to the deposited ECM. Culture supernatants were analysed at multiple time points to assess bone turnover, with increasing levels of C-terminus procollagen-I (CICP) and decreasing phosphate concentrations indicating matrix deposition, and increasing levels of N-telopeptides of collagen type-I (NTX) and raised phosphate concentrations and TRAP activity indicating matrix resorption (Figure 9). When replacing exogenous supplementation of RANKL and M-CSF with Vitamin D_3_ co-cultures still underwent osteoclastogenesis, resulting in self-regulation of the model (Papadimitropoulos et al., 2011). Sequentially monitoring key markers in the culture supernatants to assess bone turnover is similar in principle to the longitudinal imaging approaches performed by Ruggiu et al. (2014) and Rubert et al. (2017). These non-invasive and non-destructive techniques both allow the same culture to be examined at multiple time points, reducing the total number of cultures required. Ideally, these two approaches would be combined, allowing both the amount and distribution of bone turnover to be synergistically monitored.

Although scaffolds provide a 3D environment and physical cues for cells, the materials used to fabricate them are often dissimilar to natural bone tissue. Additionally, scaffolds may be doped with bioactive factors such as BMPs. Although beneficial for tissue engineering, the presence of these foreign, unrepresentative materials and factors may be obstructing the investigation of the actual sequence of cellular events that occur *in vivo* during remodelling. With this impetus, Clarke, et al., attempted to create a scaffold-free three-dimensional *in vitro* bone model by forming tissue aggregates in a rotational bioreactor. Tissue constructs were formed by culturing human osteoblasts and MNCs at a 2:1 ratio in media supplemented with exogenous RANKL and M-CSF with the rotational speed varied to alter initial aggregate size and keep the aggregate in free-fall for up to 21 days. Mineralised, solid to the touch aggregates up to 4 mm could be formed after 3 weeks. These contained a mineralised core with structures that resemble trabeculae which contained embedded cells that express sclerostin, indicating they may have become osteocytic. Surrounding the core, a morphologically different perimeter that contained active osteoblasts and osteoclasts expressing osteocalcin and TRAP, respectively, was apparent and appeared to have resorption pits. BMP-2, -4, and -7 expression was also detectable, as you would expect with remodelling bone *in vivo* (Clarke et al., 2013).

Penolazzi, et al., also utilised a rotational bioreactor in their *in vitro* remodelling system to simulate the jawbone microenvironment in the study of osteonecrosis of the jaw (ONJ), a rare side effect associated with bisphosphonate therapy. They extracted human osteoblasts from either healthy donors or patients undergoing treatment for ONJ and co-cultured them with human MNCs either indirectly or directly without exogenous RANKL and M-CSF. Indirect cultures were performed using well inserts. Direct co-cultures were either static or dynamic. Direct-static cultures were performed by generating spheroids of osteoblasts and MNCs in a 1:2 ratio on agarose-coated well plates. Direct-dynamic aggregates were formed in a rotational bioreactor at the same ratio. Indirect co-culture generated multinucleated, TRAP and CTSK positive osteoclasts, indicating the production of sRANKL. Static and dynamic direct cultures had no difference in cell viability. As with Clarke et al. aggregates within the rotational reactor had a much more defined, better organised structure with three distinct regions. Osteoblast markers OPN, OSX, Runx2, and ARS were all higher in the dynamic culture, as were osteoclast markers TRAP and CTSK. Osteoblasts from ONJ patients were lower in quality but still able to form mineralising, TRAP positive aggregates (Penolazzi et al., 2016).

The same group used this rotational bioreactor system to explore the effect of menaquinone-4 (MK-4), a member of the vitamin K2 family that can regulate calcium homeostasis and may have an anabolic effect on bone formation. Human amniotic fluid MSCs (hAFMSCs) were co-cultured with human MNCs in a 2:1 ratio without exogenous RANKL and M-CSF. In conventional 2D and dynamic 3D monocultures of hAFMSCs, 10 μM of MK-4 significantly increased mineralisation and ALP, RUNX2, OC, COL-1, and OPN mRNA expression without affecting cell viability. In dynamic 3D co-culture hAFMSCs supported osteoclastogenesis without exogenous factors, again in the outer perimeter of the aggregate. In the presence of MK-4, there were significantly fewer TRAP positive osteoclasts formed and a significant increase in mineralised matrix deposition (Mandatori et al., 2017).

Young et al. explored how the surface features of the substrate can influence bone remodelling *in vitro* by performing co-cultures on a polycarbonate surface either with or without their previously developed “NSQ50” nanotopography. This disordered but controlled surface has been demonstrated to induce osteoblast differentiation from MSCs with similar efficacy to traditional osteogenic media (Dalby et al., 2007). Human bone marrow was aspirated and separated into MSCs and haematopoietic cells. MSCs were cultured on the substrates for 1 week before the addition of the haematopoietic cells, and then cultures were maintained for a further 3 weeks with no exogenous osteoclastogenic supplements. After 21 days, large, TRAP positive, multinucleate osteoclasts with actin rings were visible on both substrates, as well as smaller TRAP positive mononuclear macrophages. There were no significant differences in osteoclastogenesis between planar and NSQ50 substrates as quantified by microscopy and TRAP, OSCAR, and CTSK mRNA expression. However, the patterned substrates stimulated increased bone mineral deposition over the planar as demonstrated by alizarin red S and osteopontin staining. RANKL, OPG, and IL-6 expression were significantly increased on the NSQ50 surfaces but by equal amounts, maintaining the RANKL:OPG ratio (Young et al., 2015). The increase in osteoblast activity and mineral production without a subsequent increase in osteoclast activity suggests that certain nanotopographies can be selectively bioactive, influencing only one cell type. Furthermore, this work has potential applications in implant osseointegration and biomaterials that can stimulate bone restoration in patients with osteoporosis.

Healthy bone remodelling requires a delicate balance between formation and resorption by BMUs. These require a blood supply which is essential for delivery of nutrients, precursors and waste removal. However, due to the complexity of angiogenesis *in vitro*, this aspect of remodelling is often overlooked. We know that the microvascular cells at sites of bone turnover can influence osteoclastogenesis, and therefore including vascularisation is essential in the attempt to mimic remodelling *in vitro* (Collin-Osdoby et al., 2001). To address this, Bongio, et al., tetra-cultured human umbilical vein endothelial cells (HUVECs), MSCs, MSCs differentiated into osteoblasts, and human MNCs within collagen/fibrin hydrogels incorporated with calcium phosphate nanoparticles in media supplemented with exogenous RANKL and M-CSF. Formation of microvessels was confirmed in hydrogels in co-cultures of HUVECs and MSCs. Monocultures of osteoblasts expressed raised proliferation, ALP activity and mineralisation over time, and monocultures of osteoclasts became TRAP positive and were able to resorb the matrix, releasing phosphate into the media. Comparing vascularisation in HUVEC-MSC vs tetra-culture, the presence of osteoblasts and osteoclasts appeared to diminish overall hydrogel vascularisation, with fewer but longer microvessels in the network. MSC cells appeared to differentiate into mural cells to support the vascular network. Comparing osteoblast/osteoclast co-cultures to the tetra-culture, the latter enhanced osteoblast and osteoclast differentiation over co-cultures, as indicated by ALP and TRAP activity and phosphate release (Bongio et al., 2016). The presence of MSCs and HUVECs within the culture positively influenced osteogenic and osteoclastic differentiation, with all four cell types synergistically influencing each other.

## Disease-orientated models

Simpler, two-dimensional co-cultures of osteoblasts and osteoclasts can help us elucidate factors that regulate the remodelling process, and by moving to more physiologically relevant three-dimensional systems we have seen the creation of increasingly complex *in vitro* models of the entire process. These will become invaluable in understanding how this intricate process occurs, as well as for evaluating potential new therapeutics and implants. However, such models also have the capacity to be adapted to study various pathologies, including cancer, osteoporosis and dental disorders.

### Cancer

Multiple myeloma is a type of cancer characterised by accumulation of plasma cells in the bone marrow and formation of osteolytic lesions due to greater osteoclast activity and reduced osteoblast activity (Giuliani et al., 2014). BRCs typically separate the BMU from the bone marrow to regulate the microenvironment and tightly control osteoblast-osteoclast coupling and therefore ensure balanced remodelling (Eriksen, 2010). However, in biopsies from patients with MM areas of uncoupled and therefore excessive resorption, which results in the formation of osteolytic lesions, have compromised BRCs which permit the passage of MM cells. Uncompromised areas had normal bone remodelling as the BRCs acted as a barrier to the cancerous cells (Andersen et al., 2009). To elucidate whether the formation of these lesions was indeed due to compromised BRCs, Anderson et al. attempted to create an *in vitro* model of the scenario. They utilised a confluent G_0_-arrested monolayer of MC3T3-E1 to simulate the BRC. Direct contact with OPM2 MM decreased the surface area covered by the MC3T3-E1, whereas indirect co-culture using a well insert had no effect. Conversely, direct co-culture with human MNC-derived osteoclasts increased the area covered (Andersen et al., 2010). This indicates that MM cells may be able to disrupt the BRC in direct cell contact.

Prostate cancer (PCa) is one of the most common cancers and has a high mortality rate due to the development of hematogenous metastases. Approximately 90% of these occur within bone, with 85–100% of patients who die from prostate cancer having bone metastases (Bubendorf et al., 2000; Carlin and Andriole, 2000; Pentyala et al., 2000). To better understand how PCa cells interact with the tissue, Nordstrand et al. developed an *in vitro* co-culture model where monolayers of either PC-3, an osteolytic human PCa cell line, or LNCaP, a human PCa cell line with a mixed/osteoblastic phenotype, were cultivated beneath a freshly harvested murine calvarial bone that still contained osteoblasts and osteoclasts. PC-3 upregulated CTSK, TRAP, MMP-9, and RANKL mRNA expression whilst inhibiting OPG, ALP and osteocalcin expression, causing a negative bone balance by increasing the RANKL:OPG ratio and decreasing osteoblast activity. In contrast to the osteolytic activity of PC-3, LNCaP raised ALP and osteocalcin expression and had no significant effect on the RANKL:OPG ratio in comparison to control calvarial cultures, indicating a small shift toward a positive bone balance (Nordstrand et al., 2009). By utilising *ex vivo* tissue in the co-culture the natural heterogeneity of the cell population in the bone tissue was maintained. This results in an *in vitro* model that can be used to study the interaction between PCa cells and bone. Li et al. also utilised PC-3 and C4-2B, a subline of LNCaP, to examine how PCa metastases influence remodelling. In both cell lines, TGF-β heightened RANKL expression and RAW264.7 differentiation, indicating PCa cells can induce osteoclastogenesis (Li et al., 2012).

Breast cancer (BCa) is another very common cancer that regularly develops bone metastases. These metastases cause a negative bone balance by increasing osteoclast activity. This releases cytokines and growth factors from the resorbed bone, which in turn stimulates cancer cell proliferation, which further exacerbates the resorption. This vicious cycle results in significant bone loss, pain and morbidity (Chen et al., 2010; Desantis et al., 2013). To investigate this, Krishnan, et al., introduced metastatic breast cancer cells to an *in vitro* bone remodelling model. Using a bioreactor they developed for long-term (< 10 months) osteoblast culture, they maintained MC3T3-E1 for 60 days before the addition of pre-osteoclasts harvested from murine bone marrow (Krishnan et al., 2010). MDA-MB-231-GFP BCa cells were added to the co-culture after a further 10 days. Cultures were maintained in media containing exogenous RANKL and M-CSF. After 60 days, the MC3T3-E1 had created and become embedded in a thick, collagenous ECM. After a further 21 days of osteoclast culture, multinucleated, TRAP positive osteoclasts that resorbed the ECM and formed pits were visible. Subsequent addition of new MC3T3-E1 resulted in the refilling of the resorbed areas, completing the remodelling process. After the initial 60 days, the ECM was 20 μm thick. This was reduced to 16.5 μm by the addition of osteoclasts and 14.5 μm by osteoclasts and BCa cells. The metastatic cells penetrated the ECM and formed osteoclasts-BCa aggregates, as well as increasing osteoclastogenesis and downregulating osteoblast differentiation in comparison to co-cultures (Krishnan et al., 2014, 2015). This model without the BCa cells includes the major processes of remodelling, albeit requiring the addition of new osteoblast pre-cursors, and provides a way of studying the process over long time periods. The addition of BCa cells provides a simplified platform for the study of how the major cellular constituents of breast cancer metastases interact.

The cannabinoid type 2 (CB2) receptor has been implicated with regulating tumour growth and bone remodelling. By agonising this receptor with JWH133 or HU308, Sophocleous, et al., determined that the growth of three BCa cell lines could be inhibited, but that the agonists have no effect on the proliferation of murine calvarial osteoblasts or bone marrow-derived osteoclasts. In co-cultures of the bone cells, conditioned media from the cancer cells upregulated osteoclastogenesis in comparison to untreated controls; however, treatment with the CB2 agonists further enhanced osteoclast formation by increasing the RANKL:OPG ratio. Treatment of osteoclast monocultures with the agonists and conditioned media raised osteoclast formation, TRAP and CTSK expression and resorption in comparison to conditioned media alone (Sophocleous et al., 2015). These findings indicate that although CB-2 activation has been shown to supress cancer cell proliferation and tumour growth at higher concentrations, lower concentrations enhanced osteolysis in this study, and therefore CB-2 inhibition may protect the skeleton in cases of BCa metastases (Lozano-Ondoua et al., 2013).

Trichostatin A (TSA) is an antibiotic that acts as an inhibitor of histone deacetylase enzymes that regulate chromatin remodelling and transcription activity, rendering it a potent anticancer drug. In co-cultures of murine osteoblasts and red blood cell free murine bone marrow cells, 10 nM TSA significantly reduced osteoclast formation but it did not alter the RANKL:OPG ratio. Instead, it acts directly on osteoclast precursors by downregulating c-fos, a transcription factor essential in osteoclastogenesis. *In vivo*, they found TSA can mitigate IL-1 induced bone loss, indicating this drug may also have potential in reducing inflammatory bone loss (Kim et al., 2009).

### Dental disorders

Cleidocranial dysplasia (CCD) is a congenital disorder that affects bone and tooth development due to mutations in the RUNX2 gene. For a tooth to erupt a path must be cleared through the bone above via resorption. Eruption is delayed in patients with CCD, therefore Lossdörfer et al. invesitgated whether this was due to PDL cells from CCD pateints having reduced capability to induce osteoclastogenesis. Human PDL cells from healthy of CCD patients were co-cultured with RAW264.7 in a 1:1 ratio. Vitamin D_3_ increased the RANKL:OPG ratio in both healthy and diseased PDL cells. Conditioned media from healthy PDL cells produced significantly more TRAP positive, multinucleated osteoclasts. In direct co-culture, PDL cells from CCD patients reduced TRAP and CTSK expression in comparison to healthy PDL cells (Lossdörfer et al., 2009). Yan et al. also investigated the delayed eruption of teeth in CCD by co-cultruing primary human dental pulp cells (DPCs) from healthy or CCD patients with hPBMCs. ALP expression and formation of mineralised nodules was reduced in CCD DPCs. In co-culture, TRAP, CTSK, and MMP-9 exression were all reduced in comaprison to healthy DPCs due to a 92% reduction in the RANKL:OPG ratio (Yan et al., 2015). The findings of Wang, et al., agree with both these studies. They co-cultured healthy or CCD patient dental follicle cells (DFCs) with hPBMCs, finding that diseased cells had a reduced capability to induce osteoclast formation through a reduction in the RANKL:OPG ratio. However, Vitamin D_3_ was only able to increase the ratio in DFCs from healthy donors as its stimulation of RANKL production is mediated prinicpally by a RUNX2 deppendent pathway (Wang et al., 2016). These data combine to indicate that the primary teeth retention associated with CCD may be due to a reduced capacity of osteoblast-like dental cells to induce osteoclastogenesis and create a path for teeth to emerge through.

### Testing of anabolic therapeutics

Osteoporosis is the most common metabolic bone disorder but as it stands there is no *in vitro* model for the study of the disease. Despite this, *in vitro* models of remodelling can be used to study potential new anabolic therapeutics for the disorder. Icariin is a phytoestrogen and a flavonoid in *Herba epimedii* that can stimulate bone formation and inhibit osteoclastogenesis (Huang et al., 2007). Liu et al. investigated whether it can have a synchronised duel effect on osteoblasts and osteoclasts in a direct co-culture of murine MSCs and RAW264.7 with exogenous RANKL and M-CSF and ovarian follicular granulosa cells (GC) in a well insert above. Co-cultures had greater ALP staining in comparison to murine OB mono-cultures, indicating osteoclastic upregulation of osteoblasts. This was further amplified when GC cells were present, and the co-culture raised the oestradiol production of the GC cells. There was no change in the amount of TRAP positive osteoclast formation in co-culture over osteoclast monoculture. Icariin was compared to common osteoporosis drugs to evaluate its efficacy. In co-culture, alendronate, a bisphosphonate, reduced both TRAP and ALP activity and PTH increased both TRAP and ALP activity. However, Icariin reduced TRAP and raised ALP activity. A similar effect was seen when substituting RAW264.7 for murine peripheral blood monocytes (Liu et al., 2015). These findings in combination with a recent ovariectomy (OVX) study indicate its potential as an anabolic therapeutic for osteoporosis (Wang et al., 2017).

Semaphorins are a class of membrane-bound or secreted proteins involved in osteoclast-osteoblast communication. Osteoclast-derived semaphorin 4D (sema4D) binds to its receptor Plexin-B1 on osteoblasts, inhibiting bone formation (Negishi-Koga et al., 2011). Therefore, it has the capability to regulate bone turnover, and overexpression of sema4D is observed in osteoporosis. Zhang et al. utilised siRNA to interfere with sema4D and create a targeted drug delivery system. By applying the siRNA to co-cultures of murine osteoblasts with bone-marrow derived osteoclast precursors, they found that application of the siRNA does not influence osteoclast number or function, but ALP, COL-1, and osteocalcin mRNA expression and mineralised matrix formation are increased in silenced cultures. When used *in vivo* in OVX mice, regular administration of the siRNA had similar results to that observed *in vitro* by significantly increasing the number of active osteoblasts and total bone volume, indicating sema4D silencing as a potential therapeutic option for osteoporotic patients (Zhang et al., 2015).

## Conclusions

An established, human-based *in vitro* model of bone remodelling is an exciting prospect due to the range of benefits it can provide. There is currently a poor translation of pre-clinical efficacy in animal models to human trials, meaning that there is a need for an alternative method of screening and evaluating new therapeutics for metabolic bone disorders. Such a model could provide a platform suitable for this that would have reduced financial and ethical costs in comparison to *in vivo* models. Furthermore, it could be utilised in the study of diseases that also affect the skeleton, such as metastatic cancers and dental disorders. Finally, it could improve our understanding of how materials will behave once implanted into the body, as well as the remodelling process itself.

To date, a range of *in vitro* studies have helped elucidate the mechanisms and biological factors by which our bones remodel. When combined with the recent advances in bone tissue engineering, this has given rise to increasingly complex systems that to some extent can mimic remodelling *in vitro*. However, despite creating functional three-dimensional systems, we are not yet in a position where the remodelling process *in vitro* can be truly recreated. Perhaps the greatest limitation of much of work performed thus far is the reliance on the addition of exogenous RANKL to the co-cultures. When diseases such as osteoporosis disrupt our bone balance, it is predominantly through an alteration in the RANKL:OPG ratio. Therefore, when this mechanism is overridden by adding RANKL exogenously, the possibility of utilising the resulting system in the study of these diseases is removed. Superficially, this limitation seems simple to overcome—simply utilise the osteoblast-lineage cells endogenous ability to produce RANKL. Regrettably, it is not as straightforward this. Although there are a small handful of studies that have successfully exploited this (Lossdörfer et al., 2009, 2011; Tortelli et al., 2009; Heinemann and Heinemann, 2011; Penolazzi et al., 2016; Mandatori et al., 2017); the majority have found that without exogenous addition osteoclastogenesis will not occur. This could be for two reasons; either RANKL is not being produced or such an excess of OPG is being co-synthesised that the resulting ratio inhibits osteoclast differentiation. Considering the first possibility, studies where cells produced sufficient endogenous RANKL had one thing in common—they were all primary cells. Therefore, it could be that its synthesis is highly donor specific. Alternatively, it could be that some essential factor is missing from the media. However, the general composition is highly similar making this unlikely, making it more likely that it is some factor present or absent in the serum. Whether either of these are the case, or whether it is the result of excessive OPG production, it is clear that a cell source capable of synthesising RANKL reproducibly and a defined, serum-free media composition are essential for a robust *in vitro* model to be developed.

**Figure 10:**
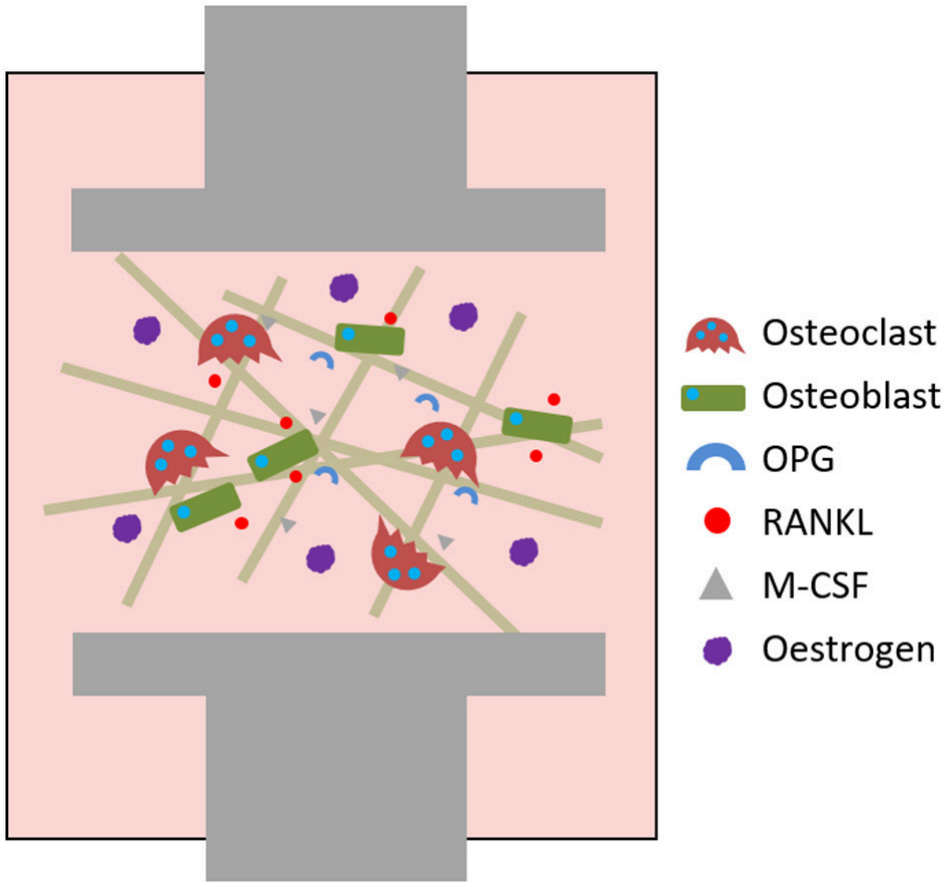
Requirements for a robust *in vitro* model of bone remodelling. A 3D co-culture of osteoblast- and osteoclast-lineage cells where the osteoblastic component are capable of endogenously producing RANKL, M-CSF and OPG. The model is cultured in a defined, serum-free medium containing physiologically relevant concentrations of important hormones, e.g., oestrogen, to permit the study of associated disorders such as postmenopausal osteoporosis. The culture can be mechanically loaded using varying force levels, for example by compression or application of fluid flow.

Two final considerations that should be accounted for in future work are mechanical stimulation and the hormones involved in remodelling, specifically oestrogen (Figure 10). Mechanical loading modulates remodelling *in vivo* and therefore should be incorporated into a representative model (Robling and Turner, 2009). This would permit the study of the effects changes in loading on bone *in vitro*. By utilising a culture media for the model that has a representative concentration of hormones knows to affect bone metabolism, there would be the possibility of modulating this to study associated disorders such as postmenopausal osteoporosis. Such an *in vitro* model of bone remodelling would perhaps have the greatest potential impact on the use of animals in musculoskeletal research.

## Author contributions

RO performed the review and wrote the manuscript. GR provided feedback and edited the manuscript.

### Conflict of interest statement

The authors declare that the research was conducted in the absence of any commercial or financial relationships that could be construed as a potential conflict of interest. The reviewer RS and handling Editor declared their shared affiliation.
